# IκBζ controls IL-17-triggered gene expression program in intestinal epithelial cells that restricts colonization of SFB and prevents Th17-associated pathologies

**DOI:** 10.1038/s41385-022-00554-3

**Published:** 2022-08-24

**Authors:** Soh Yamazaki, Naohiro Inohara, Masaki Ohmuraya, Yousuke Tsuneoka, Hideo Yagita, Takaharu Katagiri, Takashi Nishina, Tetuo Mikami, Hiromasa Funato, Kimi Araki, Hiroyasu Nakano

**Affiliations:** 1 Department of Biochemistry, Toho University School of Medicine, 5-21-16 Omorinishi, Ota-ku, Tokyo, 143-8540 Japan; 2 Department of Pathology, University of Michigan Medical School, Ann Arbor, MI 48109 USA; 3 Department of Genetics, Hyogo College of Medicine, 1-1, Mukogawa-cho, Nishinomiya, Hyogo, 663-8501 Japan; 4 Department of Anatomy, Toho University School of Medicine, 5-21-16 Omorinishi, Ota-ku, Tokyo, 143-8540 Japan; 5 Department of Immunology, Juntendo University Graduate School of Medicine, Bunkyo-ku, Tokyo, 113-8421 Japan; 6 Department of Pathology, Toho University School of Medicine, 5-21-16 Omorinishi, Ota-ku, Tokyo, 143-8540 Japan; 7 International Institutes for Integrative Sleep Medicine (WPI-IIIS), University of Tsukuba, 1-1-1 Tennodai, Tsukuba, Ibaraki 305-8575 Japan; 8 Division of Developmental Genetics, Institute of Resource Development and Analysis, Kumamoto University, 2-2-1, Honjo, Kumamoto, 860-0811 Japan; 9 Center for Metabolic Regulation of Healthy Aging, Kumamoto University, 1-1-1, Honjo, Kumamoto, 860-8556 Japan

## Abstract

Control of gut microbes is crucial for not only local defense in the intestine but also proper systemic immune responses. Although intestinal epithelial cells (IECs) play important roles in cytokine-mediated control of enterobacteria, the underlying mechanisms are not fully understood. Here we show that deletion of IκBζ in IECs in mice leads to dysbiosis with marked expansion of segmented filamentous bacteria (SFB), thereby enhancing Th17 cell development and exacerbating inflammatory diseases. Mechanistically, the IκBζ deficiency results in decrease in the number of Paneth cells and impairment in expression of IL-17-inducible genes involved in IgA production. The decrease in Paneth cells is caused by aberrant activation of IFN-γ signaling and a failure of IL-17-dependent recovery from IFN-γ-induced damage. Thus, the IL-17R–IκBζ axis in IECs contributes to the maintenance of intestinal homeostasis by serving as a key component in a regulatory loop between the gut microbiota and immune cells.

## Introduction

The maintenance of intestinal eubiosis is essential for achieving intestinal health, including the prevention of fatal infections and inappropriate immune responses.^1,2^ Intestinal epithelial cells (IECs) play pivotal roles in controlling the gut microbiota.^3–6^ Among IECs, Paneth cells release anti-microbial proteins, such as lysozymes and α-defensins, and goblet cells produce mucins, principal components of the intestinal mucosa. IECs also transport IgA into the lumen through transcytosis using polymeric immunoglobulin receptor (pIgR).^7,8^ These functions are regulated by various cytokines produced by immune cells in the lamina propria and the epithelium.^4,5^

Interleukin (IL)-17 is a multi-functional cytokine that primarily serves as an elicitor of inflammation, but is also known to contribute to the maintenance of tissue homeostasis.^9–11^ The pro-inflammatory role of IL-17 in target cells is exerted via activation of NF-κB and MAP kinases, leading to the production of chemokines and cytokines. IL-17 is constitutively synthesized by several cell types, including Th17 cells, γδT cells, and group 3 innate lymphoid cells (ILC3s), all of which are abundant in the gastrointestinal tract.^12–16^ Thus, IL-17 is presumably indispensable for intestinal homeostasis, but detailed mechanisms have not been fully elucidated. The complexity of the role of IL-17 in the intestine is exemplified by aggravation of colitis after administration of anti-IL-17 antibody in clinical trials,^17,18^ whereas the antibody is effective in other inflammatory diseases, including psoriasis and ankylosing spondylitis.^11,19,20^

The NF-κB-binding protein IκBζ (encoded by *Nfkbiz* and *NFKBIZ* in mice and humans, respectively) is a nuclear factor that activates a subset of NF-κB target genes.^21,22^ Although IκBζ was identified as a transcriptional regulator in innate immune responses, it is also involved in other physiological processes, such as the production of IFN-γ in natural killer (NK) cells,^23^ the development of Th17 cells,^24^ and the maintenance of the facial skin homeostasis.^25^ Furthermore, polymorphisms in *NFKBIZ* have been associated with human pathologies, including invasive pneumococcal disease,^26^ psoriasis,^27^ and inflammatory bowel disease.^28–30^ In vitro studies have shown that expression of IκBζ is upregulated following NF-κB-activating stimuli, such as Toll-like receptor (TLR) ligands, IL-1β, or IL-17,^31^ but the physiological role of IκBζ in response to these stimuli remains elusive. In addition, although IκBζ preferentially binds to the NF-κB p50 subunit rather than other NF-κB family members,^21^ the in vivo aspects of the cooperation between IκBζ and p50 is also mostly unknown.

In the present study, we demonstrate that deletion of IκBζ in IECs results in the marked expansion of segmented filamentous bacteria (SFB). The expansion of SFB leads to enhanced development of Th17 cells and aggravation of inflammatory diseases. The dysregulation of microbiota is attributable to impaired IgA secretion and loss of Paneth cell integrity. In small intestinal organoids, IκBζ is required for IL-17A-induced upregulation of a set of genes including *Pigr*. Even though IL-17 signaling is dispensable for the development of Paneth cells in organoids under standard culture conditions, stimulation of organoids with IL-17A allows efficient restoration of Paneth cells after IFN-γ-induced damage in an IκBζ-dependent manner. The decrease in Paneth cells in the intestine of IEC-specific IκBζ-deficient mice is triggered by aberrant upregulation of IFN-γ signaling as injection of anti-IFN-γ antibody attenuates the decrease in Paneth cells. Consistent with the preferential binding of IκBζ to the NF-κB p50 subunit, the expression of a similar set of genes is impaired by the lack of p50 in the small intestine of mice and IL-17A-stimulated intestinal organoids. Thus, the specific NF-κB-regulated gene program in IECs play an important role in the control of the intestinal microbiota.

## Results

### Deletion of IκBζ in IECs causes an aberrant increase in Th17 cells in the small intestine and exacerbation of inflammatory diseases

**Fig. 1:**
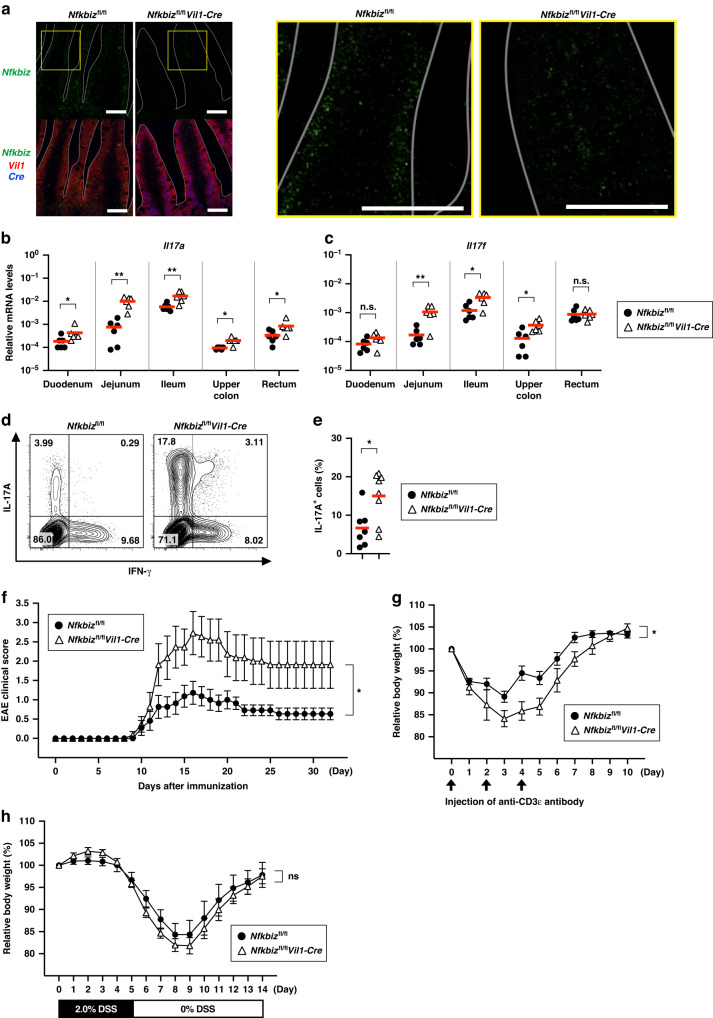
Deletion of IκBζ in IECs causes an aberrant increase in Th17 cells in the small intestine and exacerbation of inflammatory diseases. **a** A section of the jejunum was prepared from control (*Nfkbiz*^fl/fl^) or IEC-specific IκBζ-deficient (*Nfkbiz*^fl/fl^*Vil1-Cre*) mice and the expression of the indicated genes was analyzed by in situ hybridization. Magnified images of the yellow box are shown to the right. Scale bar, 50 μm. The villi are delineated by white lines. **b**, **c** The expression of *Il17a* (**b**) and *Il17f* (**c**) was determined by RT-qPCR. The mean expression levels are shown (*n* = 6 mice). **d**, **e** Lamina propria cells in the small intestine from the indicated mice were subjected to intracellular cytokine staining and analyzed by flow cytometry. The percentage of cells in each quadrant among FVD506^–^CD45^+^CD4^+^TCR^β+^ (CD4^+^ T) cells are shown (**d**). The results are representative of 7–8 independent experiments. The mean frequencies (percentages) of IL-17A^+^ cells among CD4^+^ T cells are shown (*n* = 7–8 mice per group) (**e**). **f** For induction of EAE, the indicated mice were immunized with MOG_31–55_ peptide on day 0 and injected with Pertussis toxin on days 0 and 2. Clinical scores were determined every day, and the mean clinical scores ±SEM (*n* = 11 mice per group) are shown. Data are pooled results from two independent experiments. **g** For induction of enteritis in the small intestine, the indicated mice were intraperitoneally injected with an agonistic anti-CD3ε antibody (1.0 mg/kg) on days 0, 2, and 4, and the body weight was measured every 24 h. The body weight is given as the percentage relative to the value on day 0. Results are presented as mean ± SEM (*n* = 7–8 mice per group). Data are pooled results from two independent experiments. **h** For induction of colitis, the indicated mice were administered 2.0% DSS in drinking water for 5 days, followed by regular water without DSS in subsequent days. Body weight was measured every 24 h, and is shown as the percentage relative to the value on day 0. Results are presented as mean ± SEM (*n* = 12–13 mice per group). Data are pooled results from two independent experiments. Statistical significance was determined by the Mann–Whitney *U* test (**b**, **c**, **e**) or two-way ANOVA (**f**, **g**, **h**). *p < 0.05, **p < 0.01, n.s., not significant.

IL-17A is constitutively produced in the intestine by a group of RORγt^+^ cells present in the lamina propria, including Th17 cells and ILC3 cells.^12,16^ As the expression of IκBζ is upregulated in response to IL-17A,^31^ IκBζ is expected to have an important function in the intestine. Public databases show that *Nfkbiz* is highly expressed in the gastrointestinal tract compared to other tissues (http://biogps.org/#goto=genereport&id=80859, https://www.ncbi.nlm.nih.gov/gene/80859). We therefore investigated the expression of *Nfkbiz* in different regions of the intestine in wild-type mice and observed higher levels of expression in the small intestine than in the large intestine (Supplementary Fig. 1a). Fractionation of ileal tissue after EDTA treatment revealed that *Nfkbiz* was expressed in the intestinal epithelium as well as the lamina propria (Supplementary Fig. 1b). Because intestinal epithelial cells (IECs) play key roles in IL-17-related biology,^4,10^ we attempted to elucidate the role of IκBζ in IECs in vivo. To this end, we generated IEC-specific *Nfkbiz*-deficient (*Nfkbiz*^fl/fl^*Vil1-Cre*) mice by crossing *Nfkbiz*^fl/fl^ mice (Supplementary Fig. 1c) with *Vil1-Cre* driver mice.^32^ In *Nfkbiz*^fl/fl^*Vil1-Cre* mice, expression of *Nfkbiz* was abrogated specifically in the intestinal epithelium when analyzed by a sensitive in situ hybridization technique^33^ (Fig. 1a). *Nfkbiz*^fl/fl^*Vil1-Cre* mice grew normally and did not exhibit any obvious gastrointestinal abnormalities, such as diarrhea and hematochezia, in a standard specific pathogen-free (SPF)-rearing environment.

To investigate the role of IκBζ in the IL-17-dependent physiology of the intestine, we analyzed levels of IL-17 in the intestine of *Nfkbiz*^fl/fl^*Vil1-Cre* mice. Unexpectedly, the expression of *Il17a* and *Il17f*, two principal IL-17 family genes in the gut, was markedly upregulated in the gastrointestinal tract of *Nfkbiz*^fl/fl^*Vil1-Cre* mice compared to control mice (Fig. 1b, c). As IL-17 has been reported to be produced mainly by CD4^+^ T cells (Th17 cells) in the small intestine,^12^ we examined whether the development of Th17 cells was increased in *Nfkbiz*^fl/fl^*Vil1-Cre* mice. The number of Th17 cells was markedly increased in *Nfkbiz*^fl/fl^*Vil1-Cre* mice compared to control mice (Fig. 1d, e). Although IL-17 is also produced by other cell types, including γδT cells and ILC3s,^14,16^ the majority of IL-17A was derived from CD4^+^ T cells in the small intestine of *Nfkbiz*^fl/fl^*Vil1-Cre* mice (Supplementary Fig. 2a). The expression of another Th17-related cytokine gene, *Il22*, and the IL-22-regulated anti-microbial genes *Reg3b* and *Reg3g* was also upregulated in *Nfkbiz*^fl/fl^*Vil1-Cre* mice (Supplementary Fig. 2b). As Th17 cells are responsible for the pathogenesis of various inflammatory diseases, such as experimental autoimmune encephalomyelitis (EAE),^34,35^ we induced EAE in *Nfkbiz*^fl/fl^*Vil1-Cre* mice and found that they developed more severe disease than control mice (Fig. 1f). The spinal cords of *Nfkbiz*^fl/fl^*Vil1-Cre* mice consistently contained increased number of demyelinated areas compared to those of control mice after induction of EAE (Supplementary Fig. 3a, b), while the exacerbation of the disease was not simply attributable to Th17 cytokine responses in the central nervous system (Supplementary Fig. 3c). We also observed that, when enteritis was induced in the small intestine by repeated injections of an anti-CD3ε agonistic antibody,^36,37^ *Nfkbiz*^fl/fl^*Vil1-Cre* mice lost more weight than control mice, which is indicative of increased inflammation (Fig. 1g). The aggravation of the enteritis in *Nfkbiz*^fl/fl^*Vil1-Cre* mice is consistent with a pathological role of Th17 cells in this enteritis model.^38,39^ The intestines of *Nfkbiz*^fl/fl^*Vil1-Cre* mice exhibited more severe histopathology characteristic of this enteritis, such as shortening of villi and flattened epithelium^36^ (Supplementary Fig. 3d, e). The expression of Th17-related cytokines was increased in the intestines of *Nfkbiz*^fl/fl^*Vil1-Cre* mice compared to that of control mice after induction of the enteritis (Supplementary Fig. 3f). On the other hand, in a chemically-induced colitis model by administration of dextran sulfate sodium (DSS),^40,41^ the disease progression in *Nfkbiz*^fl/fl^*Vil1-Cre* mice was comparable to that of control mice (Fig. 1h). Thus, deletion of IκBζ in IECs enhances the development of Th17 cells, thereby exacerbating inflammatory diseases.

### Lack of IκBζ in IECs causes alterations in the microbiota including marked expansion of SFB in the small intestine

**Fig. 2:**
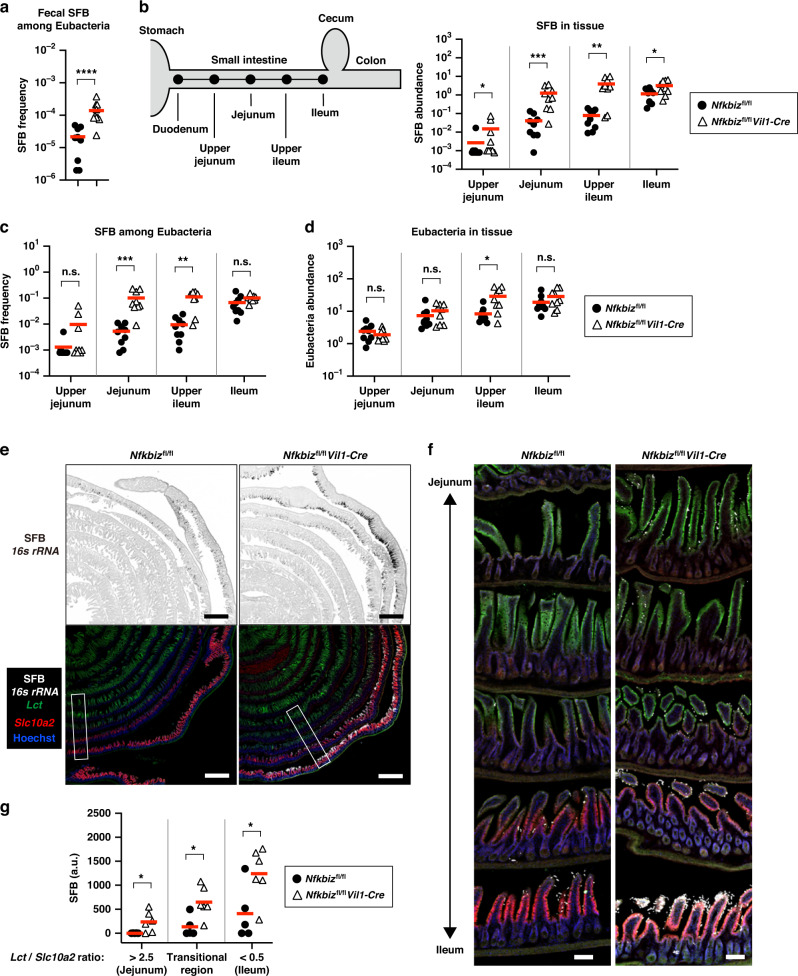
Lack of IκBζ in IECs results in marked expansion of SFB in the small intestine. **a** DNA was extracted from the feces of *Nfkbiz*^fl/fl^*Vil1-Cre* mice and co-housed gender-matched control mice (*Nfkbiz*^fl/fl^). The amount of SFB was determined by qPCR, and shown as the SFB frequency among total eubacteria. The mean values are shown (*n* = 9 mice per group). **b–d** DNA was extracted from the indicated gastrointestinal regions of *Nfkbiz*^fl/fl^*Vil1-Cre* and co-housed gender-matched control mice (*Nfkbiz*^fl/fl^). The amount of SFB in each tissue was determined by qPCR and given after normalization to *Actb* (**b**). The mean values of frequency of SFB among total eubacteria (**c**) and the amount of Eubacteria in tissue (**d**) are shown (*n* = 9 mice per group). **e** The whole small intestine from the indicated mice was fixed, and embedded after preparation of the “Swiss roll”. The cryo-section of the intestine was analyzed by in situ hybridization using the indicated probes and staining with Hoechst^Ⓡ^33342 (*n* = 5–6 mice per group). Scale bar, 1 mm. **f** The magnified images of the boxed area in (**e**) are shown. Scale bar, 100 μm. **g** The intestinal region was defined by the ratio of the florescent intensity of *Lct* to that of *Slc10a2*, and the signals for SFB in each region in (**f**) were quantified. The mean signal intensities are shown (*n* = 5–6 mice per group). Statistical significance was determined by Mann–Whitney *U* test. *p < 0.05, **p < 0.01, ***p < 0.001, n.s. not significant.

The development of Th17 cells is induced by colonization of SFB, a well-known indigenous species, at the epithelium of the small intestine.^42^ To examine whether the increase in Th17 cells in *Nfkbiz*^fl/fl^*Vil1-Cre* mice was caused by SFB, we analyzed the abundance of SFB in feces and the small intestine. The amount of SFB-derived DNA was markedly increased in the feces of *Nfkbiz*^fl/fl^*Vil1-Cre* mice compared to that of co-housed gender-matched control mice (Fig. 2a). SFB exclusively colonizes the ileum within the gastrointestinal tract of mice.^43^ The amount of SFB in the ileal tissue was moderately elevated in *Nfkbiz*^fl/fl^*Vil1-Cre* mice compared to that in control mice (Fig. 2b). Notably, the amount of SFB was strikingly increased in the upper regions of the small intestine in *Nfkbiz*^fl/fl^*Vil1-Cre* mice (Fig. 2b). The frequency of SFB among total bacteria was significantly increased in the jejunum and upper ileum of *Nfkbiz*^fl/fl^*Vil1-Cre* mice compared to control mice (Fig. 2c), and the expansion of SFB possibly resulted in an increase in the total bacteria at the upper ileum (Fig. 2d). To further investigate the distribution of SFB in the small intestine, we performed in situ hybridization using marker probes for the upper region (*Lct*) and the ileum (*Slc10a2*). In contrast to the limited localization of SFB at the ileum in control mice, SFB signal extended to the upper regions of the small intestine and was overall increased in the small intestine of *Nfkbiz*^fl/fl^*Vil1-Cre* mice (Fig. 2e–g).

**Fig. 3:**
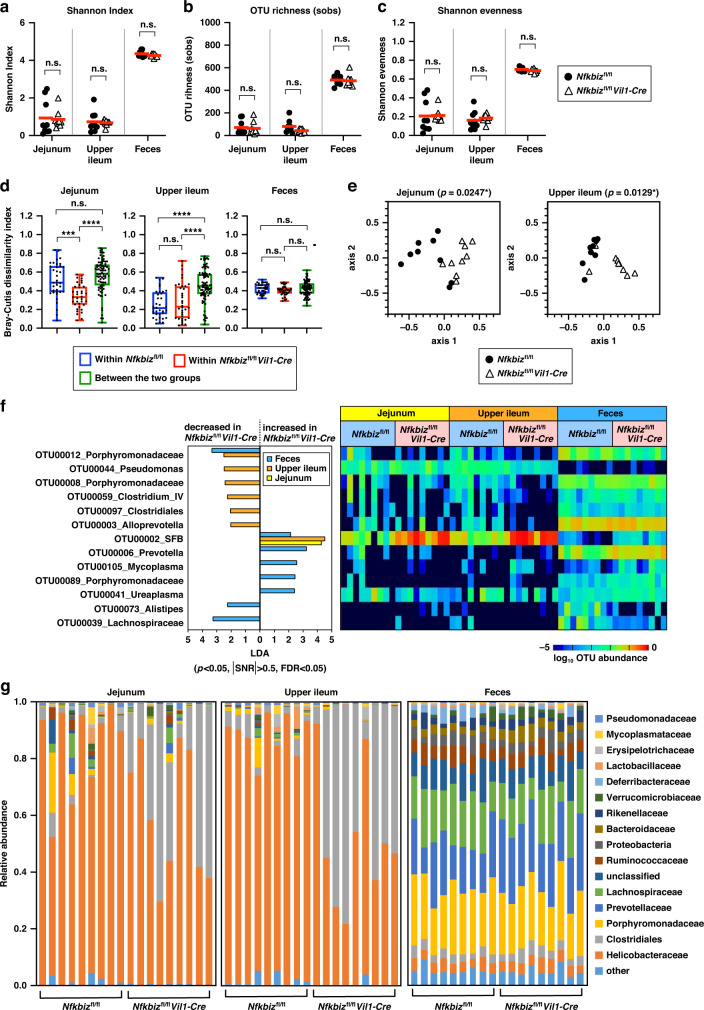
Lack of IκBζ in IECs causes drastic alteration in the microbiota in the small intestine. Bacterial composition of the jejunum, upper ileum, or feces from the indicated mice was investigated by sequencing the v4 region of the 16 S rRNA gene (*n* = 9 mice per group). **a–c** α-diversity based on the Shannon index (**a**) OTU richness (**b**), and Shannon evenness (**c**) was examined. The mean values are shown. Statistical significance was determined by Mann–Whitney *U* test**. d**, **e** β-diversity between control (*Nfkbiz*^fl/fl^) and *Nfkbiz*^fl/fl^*Vil1-Cre* mice was examined. Bray–Curtis dissimilarity indices of microbiota within each group and that between the two groups are shown as the box plot, and statistical significance was determined by Kruskal–Wallis test followed by Dunn’s multiple comparisons test (**d**). *P* values in the non-metric multi-dimensional scaling (NMDS) plot were obtained using PERMANOVA (**e**). **f** Linear discriminant analysis effect size (LEfSe) was analyzed. Differentially abundant operational taxonomic units (OTUs) are shown with linear discriminant analysis (LDA) values in LEfSe in accordance with the criteria of *p* < 0.05, positive false discovery rate (FDR) < 0.05, and modulus of signal-to-noise ratio (|SNR | ) > 0.5. **g** The composition of taxonomic families and orders was analyzed. *p < 0.05, **p < 0.01, ***p < 0.001, ****p < 0.0001, n.s., not significant.

We also analyzed the microbiome within the jejunum, upper ileum, and feces from *Nfkbiz*^fl/fl^*Vil1-Cre* mice that had been co-housed with control mice. The composition of the microbiota was determined by sequencing the v4 region of the bacterial 16 S rRNA gene using the Illumina MiSeq sequencer.^44^ The α-diversities of the microbiota in the jejunum, upper ileum, and feces were comparable between *Nfkbiz*^fl/fl^*Vil1-Cre* mice and control mice, when assessed by the Shannon index, OTU (operational taxonomic unit) richness, and Shannon evenness (Fig. 3a–c). On the other hand, β-diversity based on the Bray–Curtis dissimilarity index was significantly different in the small intestine between *Nfkbiz*^fl/fl^*Vil1-Cre* and control mice, indicating an alteration in the overall bacterial composition (Fig. 3d). We also generated non-metric multi-dimensional scaling (NMDS) plots from the Bray–Curtis dissimilarity index of individual mice and found that the microbiota in the small intestinal regions of *Nfkbiz*^fl/fl^*Vil1-Cre* mice was distinct from that of control mice (Fig. 3e). Analysis using the linear discriminant analysis effect size (LEfSe) identified SFB as an OTU that discriminates *Nfkbiz*^fl/fl^*Vil1-Cre* mice from control mice in the jejunum, upper ileum, and feces (Fig. 3f). Though several OTUs were decreased in the upper ileum of *Nfkbiz*^fl/fl^*Vil1-Cre* mice compared to control mice (Fig. 3f), their decrease in frequency may be a consequence of the marked expansion of SFB. The striking increase in SFB in the small intestine also affected the bacterial composition at the family and order levels (Fig. 3g). Although the family *Helicobacteraceae* occupied the majority of microbiota in the small intestines of control mice, the order *Clostridiales*, to which SFB belong, accounted for the largest fraction in *Nfkbiz*^fl/fl^*Vil1-Cre* mice (Fig. 3g). Thus, a lack of IκBζ in IECs led to drastic alterations in the microbiota in the small intestine especially with marked expansion of SFB.

### Deletion of IκBζ impairs expression of microbe-controlling genes in IECs of the small intestine

**Fig. 4:**
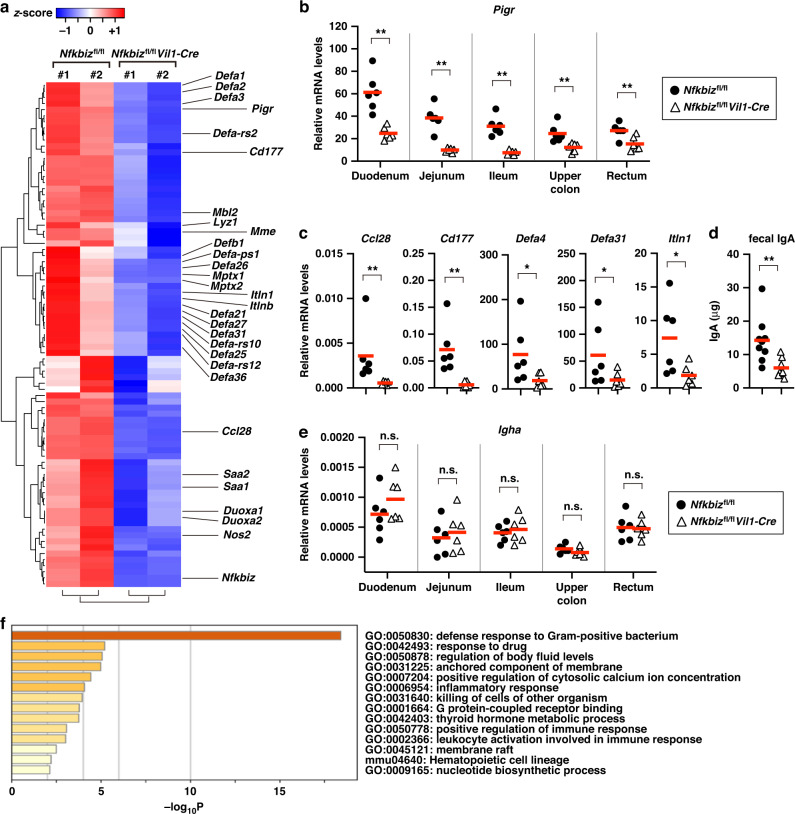
Deletion of IκBζ impairs expression of microbe-controlling genes in IECs of the small intestine. **a** Total RNA was extracted from the ilea of two pairs of *Nfkbiz*^fl/fl^*Vil1-Cre* and co-housed gender-matched controls (*Nfkbiz*^fl/fl^), and the gene expression profiles were analyzed by microarray analysis (*n* = 2 mice per group). A heat map of 83 genes down-regulated in *Nfkbiz*^fl/fl^*Vil1-Cre* mice (<50% of control mice in both pairs) is shown. **b**, **c** Total RNA was extracted from the indicated intestinal regions (**b**) or the ileum (**c**), and the expression of the indicated genes was analyzed by RT-qPCR. The mean expression levels are shown (*n* = 6 mice per group). **d** Total fecal proteins were extracted from the indicated mice and the amount of IgA was determined by ELISA. The IgA amounts are shown as per 100 μg of total fecal proteins. The mean values are shown (*n* = 9 mice per group). **e** Expression of *Igha* in the indicated intestinal regions was analyzed as in (**b**). **f** Pathway and process enrichment analysis of the 83 down-regulated genes in *Nfkbiz*^fl/fl^*Vil1-Cre* mice. Statistical significance was determined by the Mann–Whitney *U* test (**b**–**e**). *p < 0.05, **p < 0.01, n.s., not significant.

We conducted transcriptome analysis on the small intestines of *Nfkbiz*^fl/fl^*Vil1-Cre* mice to determine if the alteration of gut microbiota in *Nfkbiz*^fl/fl^*Vil1-Cre* mice was caused by a defect in gene regulation in IECs. A microarray gene chip analysis revealed that the expression of multiple genes was decreased in the ilea of *Nfkbiz*^fl/fl^*Vil1-Cre* mice compared to control mice (Fig. 4a). The downregulated genes in *Nfkbiz*^fl/fl^*Vil1-Cre* mice included those encoding *Pigr*, the epithelial chemokine CCL28 (*Ccl28*), and Paneth cell-associated proteins, such as lysozymes (*Lyz1* and *Lyz2*) and α-defensin family members (*Defa* genes) (Fig. 4a–c). The expression of *Pigr* was decreased not only in the ileum, but also in other regions of the gastrointestinal tract of *Nfkbiz*^fl/fl^*Vil1-Cre* mice (Fig. 4b). Consistent with the decreased expression of *Pigr* and *Ccl28*, fecal IgA levels were greatly reduced in *Nfkbiz*^fl/fl^*Vil1-Cre* mice compared to control mice (Fig. 4d). Expression of *Igha*, which encodes IgA, was not decreased in the intestine of *Nfkbiz*^fl/fl^*Vil1-Cre* mice compared to control mice (Fig. 4e), suggesting that the decrease in fecal IgA was caused by a defect in pIgR-dependent transepithelial transport of IgA. Pathway and process enrichment analysis by Metascape (https://metascape.org/) revealed that the genes downregulated in the small intestine of *Nfkbiz*^fl/fl^*Vil1-Cre* mice are associated with anti-bacterial functions (Fig. 4f). Thus, the dysbiosis in *Nfkbiz*^fl/fl^*Vil1-Cre* mice was likely caused by the impaired expression of multiple microbe-controlling genes.

### IκBζ is required for IL-17-induced gene expression in IECs

**Fig. 5:**
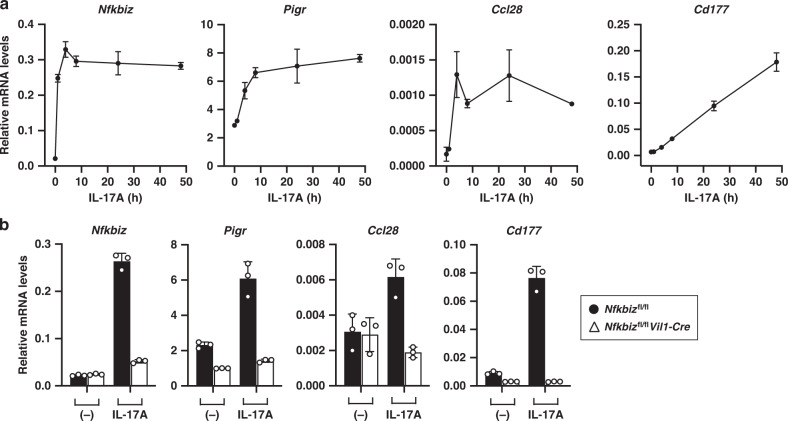
IκBζ is required for IL-17-induced gene expression in IECs. **a** Organoids were prepared from the small intestine of wild-type mice, and stimulated with IL-17A (20 ng/ml) for the indicated periods. Expression of the indicated genes was analyzed by RT-qPCR. The results are presented as the mean ± SD of triplicates and are representative of organoids from three mice. **b** The organoids from control (*Nfkbiz*^fl/fl^) or *Nfkbiz*^fl/fl^*Vil1-Cre* mice were unstimulated or stimulated with IL-17A for 24 h, and analyzed as in (**a**).

The role of IL-17 in the maintenance of intestinal homeostasis is in part related to the regulation of the gut microbiota.^9,10^ To investigate the impact of IL-17 on the expression of the microbe-controlling genes in IECs, we employed an in vitro organoid culture system prepared from the mouse small intestine.^45,46^ Expression of *Nfkbiz* was rapidly induced upon IL-17A stimulation of organoid cultures and sustained for at least 48 h (Fig. 5a), which is in contrast to the short duration of *Nfkbiz* expression in TLR/IL-1R responses.^21^ Among the genes downregulated in the intestine of *Nfkbiz*^fl/fl^*Vil1-Cre* mice, the expression of *Pigr*, *Ccl28*, and *Cd177* was induced after IL-17A stimulation (Fig. 5a), and the induction was dependent on IκBζ (Fig. 5b). These results indicate that, despite a large amount of IL-17 being released from the expanded Th17 cells in *Nfkbiz*^fl/fl^*Vil1-Cre* mice (Fig. 1b–e), the IECs of the mutant mice fail to express genes related to microbial control in response to IL-17 due to a lack of IκBζ.

### Deletion of IκBζ in IECs leads to decrease in Paneth cells

**Fig. 6:**
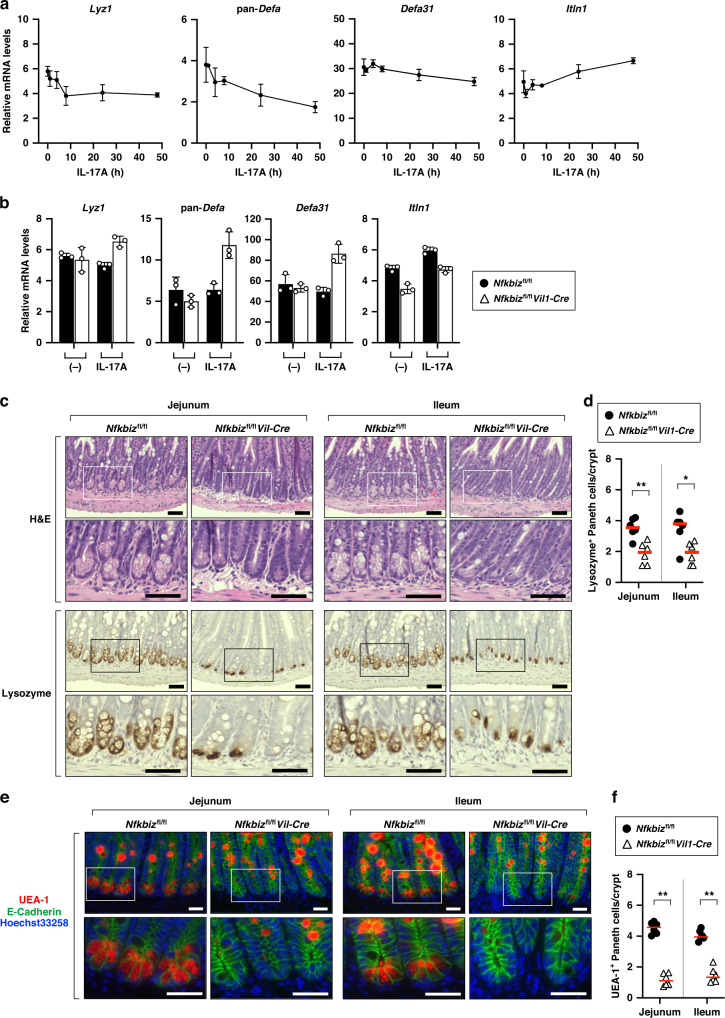
Deletion of IκBζ in IECs leads to decrease in Paneth cells. **a** Small intestinal organoids from wild-type mice were stimulated with IL-17A (20 ng/ml) for the indicated periods. Expression of the indicated genes was analyzed by RT-qPCR. The results are presented as the mean ± SD of triplicates and are representative of organoids from three mice. **b** Organoids from control (*Nfkbiz*^fl/fl^) or *Nfkbiz*^fl/fl^*Vil1-Cre* mice were unstimulated or stimulated with IL-17A for 24 h and the expression of the indicated genes was analyzed as in (**a**). **c**, **d** Tissue sections of the indicated regions of the small intestine were stained with hematoxylin-eosin (H&E) or anti-lysozyme antibody. Scale bars, 50 μm (**c**). **e**, **f** Tissue sections were stained with UEA-1, anti-E-cadherin antibody, and Hoechst 33258. Scale bars, 25 μm. (**e**). Magnified images of the box are shown to the bottom. Results are representative of six independent experiments (**c**, **e**). Lysozyme^+^ or UEA^-^1^+^ cells were counted in 50 crypts per region per mouse (*n* = 6 mice per group), and are shown as the mean number per crypt (**d**, **f**). Statistical significance was determined by the Mann–Whitney *U* test. *p < 0.05, **p < 0.01.

The Paneth cell-associated genes, including *Lyz1*, *Itln1*, and *Defa* (α-defensin) members, were also downregulated in the small intestines of *Nfkbiz*^fl/fl^*Vil1-Cre* mice (Fig. 4a, c). However, the expression of these Paneth cell-associated genes, except for *Itln1*, was not upregulated by IL-17 in small intestinal organoids (Fig. 6a). In IκBζ-deficient organoids, their expression was not abrogated, but rather upregulated after IL-17A stimulation (Fig. 6b). The expression of *Itln1* was moderately reduced in the absence of IκBζ (Fig. 6b). Given that the expression of the Paneth cell-associated genes was globally decreased in the small intestine of *Nfkbiz*^fl/fl^*Vil1-Cre* mice (Fig. 4a), we reasoned that the number of Paneth cells may be reduced in the intestine of mutant mice. Histological analysis revealed that the number of Lysozyme-expressing Paneth cells was noticeably decreased in the intestinal crypts of *Nfkbiz*^fl/fl^*Vil1-Cre* mice compared to control mice (Fig. 6c, d), which was confirmed by staining with *Ulex europaeus* agglutinin-1 (UEA-1) (Fig. 6e, f). The remaining Paneth cells in *Nfkbiz*^fl/fl^*Vil1-Cre* mice exhibited morphological abnormalities such as degeneration of granules (Fig. 6c). The defect in Paneth cells was unlikely caused by an intrinsic lack of IκBζ in Paneth cells, because deletion of IκBζ in *Lyz2*-expressing Paneth cells did not impair their integrity (Supplementary Fig. 4a, b). Thus, the maintenance of Paneth cell integrity is likely dependent on the expression of IκBζ in other type of IECs. Consistent with this, the expression of *Nfkbiz* was hardly detectable in Paneth cells among IECs (Fig. 1a and Supplementary Fig. 4c).

### The IL-17R–IκBζ axis facilitates restoration of Paneth cells from IFN-γ-induced damage

**Fig. 7:**
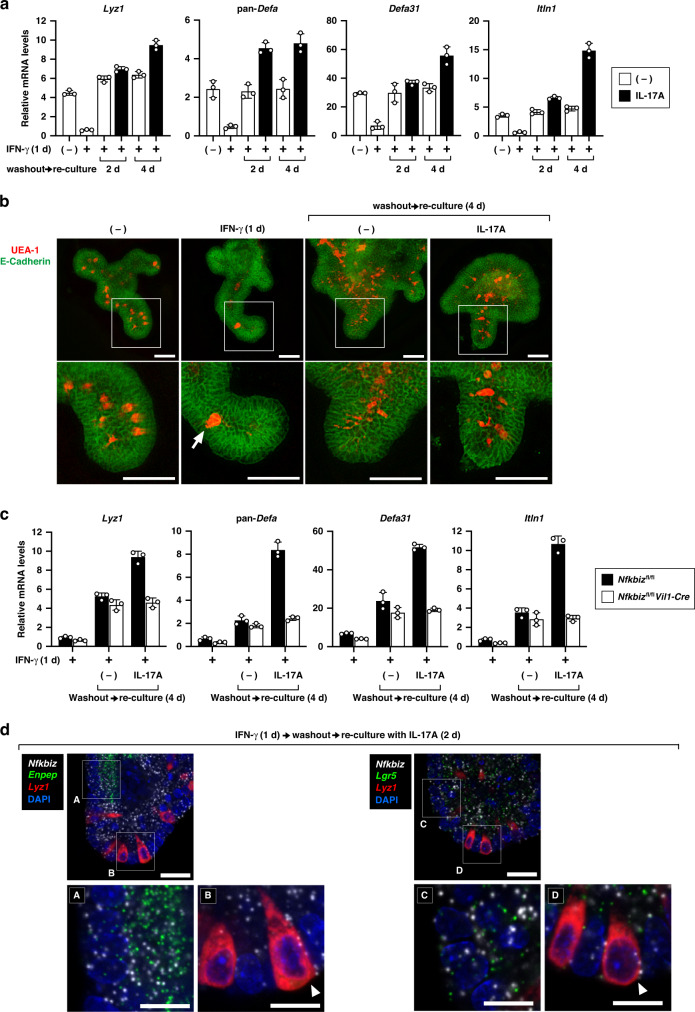
The IL-17R–IκBζ axis facilitates restoration of Paneth cells from IFN-γ-induced damage. **a** After treatment with IFN-γ (20 ng/ml) for 1 day, wild-type organoids were washed twice and then re-cultured in fresh media for 2 or 4 days in the absence or presence of IL-17A (20 ng/ml). Expression of the indicated genes was analyzed by RT-qPCR. The results are presented as the mean ± SD of triplicates and are representative of organoids from three mice. **b** Wild-type organoids treated as indicated were stained with UEA-1 and anti-E-cadherin antibody. Magnified images of the box are shown to the bottom. Results are representative of four independent experiments. The arrow indicates the release of Paneth cell granule into the crypt lumen. Scale bar, 50 μm. **c** Organoids from control (*Nfkbiz*^fl/fl^) or *Nfkbiz*^fl/fl^*Vil1-Cre* mice were treated with IFN-γ (20 ng/ml). The organoids were washed out, and re-cultured for 4 days in the absence or presence of IL-17A (20 ng/ml). Expression of the indicated genes was analyzed as in (**a**). **d** Wild-type organoids were treated as indicated, and analyzed by in situ hybridization (RNAscope) using probes specific to *Nfkbiz*, *Enpep* (an enterocyte marker), *Lyz1* (a Paneth cell marker), and *Lgr5* (an intestinal stem cell marker). Magnified images of the indicated boxes are shown to the bottom. Results are representative of two independent experiments. Scale bar, 20 μm (top) and 5 μm (bottom). The arrowheads in B and D indicate Paneth cells with the signals of *Nfkbiz* expression.

The expression of Paneth cell-associated genes was decreased in the intestines of *Nfkbiz*^fl/fl^*Vil1-Cre* mice (Fig. 4a, c) due to reduction in the number of Paneth cells (Fig. 6c–f), but this phenotype was not recapitulated in IκBζ-deficient organoids under standard culture conditions in the presence or absence of IL-17A (Fig. 6b). UEA-1^+^ Paneth cells were normally present in the organoids from the small intestine of *Nfkbiz*^fl/fl^*Vil1-Cre* mice (Supplementary Fig. 4d). We wondered whether the IL-17R–IκBζ axis is required for the maintenance of Paneth cells only under certain conditions such as during inflammation-induced Paneth cell damage. As previously reported,^47,48^ Paneth cell death was induced by IFN-γ in organoid culture, which can be monitored by quantifying the expression of Paneth cell-associated genes and staining with UEA-1 (Fig. 7a, b and Supplementary Fig. 5a). Expression of *Nfkbiz* was not decreased after IFN-γ stimulation (Supplementary Fig. 5a), which is consistent with *Nfkbiz* not being highly expressed in Paneth cells (Fig. 1a and Supplementary Fig. 4c). As reported previously,^47,48^ treatment of organoids with IFN-γ also diminished goblet cells (*Muc2*) (Supplementary Fig. 5a), whereas enterocytes (*Vil1*) were largely unchanged and the MHC class II gene (*H2-Aa*) was strongly upregulated in response to IFN-γ (Supplementary Fig. 5a). To examine the effect of IL-17A on IFN-γ-induced decrease in Paneth cells, we treated organoid cultures with the two cytokines IFN-γ and IL-17 simultaneously, and observed that IFN-γ-induced Paneth cell decrease was not attenuated by co-treatment with IL-17A (Supplementary Fig. 5b). Expression of *Nfkbiz* was induced by IL-17A even in the presence of IFN-γ (Supplementary Fig. 5b).

We noted that Paneth cells were recovered with time after IFN-γ treatment, if the organoids were washed out and re-cultured in fresh media without IFN-γ (Fig. 7a, b). Importantly, the recovery of Paneth cells was enhanced in the presence of IL-17A (Fig. 7a). The expression of *Muc2* and *Lgr5* (markers of goblet cells and intestinal stem cells, respectively) also recovered during the re-culture, but their recovery was not enhanced by IL-17A (Supplementary Fig. 5c), suggesting that IL-17A specifically promotes the recovery of Paneth cells from IFN-γ-induced damage. We further investigated the Paneth cell recovery using the organoids from *Nfkbiz*^fl/fl^*Vil1-Cre* mice, as expression of *Nfkbiz* was upregulated by IL-17A after re-culturing in fresh media (Supplementary Fig. 5c). The IL-17A-mediated enhancement of Paneth cell recovery was entirely abrogated in IκBζ-deficient organoids (Fig. 7c). Thus, the decrease in the number of Paneth cells in the small intestine of *Nfkbiz*^fl/fl^*Vil1-Cre* mice is likely a consequence of a defect in the IL-17-mediated recovery of Paneth cells after inflammation-related damages.

Although Paneth cells were largely unaffected in the small intestines of *Nfkbiz*^fl/fl^*Lyz2*^Cre^ mice (Supplementary Fig. 4a, b), the organoids from these mice exhibited a moderately impaired response to IL-17A in the recovery of Paneth cells after the IFN-γ damage (Supplementary Fig. 6a). Consistent with this, an in situ hybridization analysis using RNAscope revealed that the expression of *Nfkbiz* was observed in the recovering Paneth cells following IL-17A stimulation, while the expression of *Nfkbiz* in Paneth cells was much less than that in enterocytes and Lgr5^+^ stem cells (Fig. 7d and Supplementary Fig. 6b). IκBζ possibly mediates the role of Lgr5^+^ stem cells in the IL-17-mediated development of secretory IEC lineages including Paneth cells.^49^

### Lack of IκBζ in IECs causes aberrant activation of IFN-γ signaling in the small intestine

**Fig. 8:**
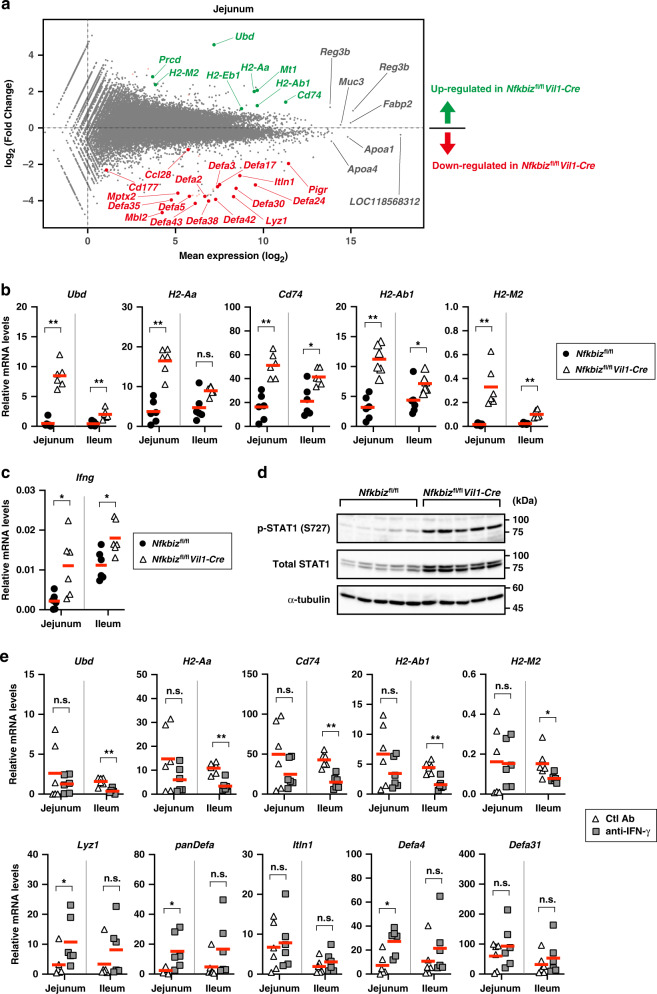
Lack of IκBζ in IECs causes aberrant activation of IFN-γ signaling. **a** Total RNA was extracted from the jejuna of *Nfkbiz*^fl/fl^*Vil1-Cre* mice and co-housed gender-matched controls (*Nfkbiz*^fl/fl^), and the gene expression profiles were analyzed by RNA-seq analysis (*n* = 3 mice per group). The MA plot is shown to visualize mean expression levels (*x*-axis) and the fold change in *Nfkbiz*^fl/fl^*Vil1-Cre* mice over control mice (*y*-axis) for each gene. Down-regulated and up-regulated genes in the jejuna of *Nfkbiz*^fl/fl^*Vil1-Cre* mice are shown in red and green, respectively. **b**, **c** Expression of the indicated genes in the jejuna and ilea was analyzed by RT-qPCR (*n* = 6 mice per group). The mean expression levels are shown. **d** Lysates from the jejuna of the indicated mice were analyzed by immunoblotting using the indicated antibodies (*n* = 5). **e** *Nfkbiz*^fl/fl^*Vil1-Cre* mice were intraperitoneally injected with an anti-IFN-γ antibody or an isotype control antibody (15 mg/kg) every 3 days, and the intestines were removed 24 h after the last injection. Expression of the indicated genes was analyzed as in (**b**) (*n* = 6 mice per group). Statistical significance was determined by Mann–Whitney *U* test (**b**, **c**, **f**). *p < 0.05, **p < 0.01, n.s., not significant.

As the expansion of SFB was more prominent at the jejunum than the ileum in *Nfkbiz*^fl/fl^*Vil1-Cre* mice (Fig. 2), we reasoned that the lack of IκBζ in IECs may have a greater impact on gene expression in the jejunum. Intriguingly, RNA-seq analysis revealed that a set of IFN-γ-inducible genes was markedly upregulated in the jejuna of *Nfkbiz*^fl/fl^*Vil1-Cre* mice in addition to the downregulation of microbe-controlling genes similarly to that observed in the ileum (Fig. 8a, b). The increase in expression of IFN-γ-inducible genes was also observed in the ilea of *Nfkbiz*^fl/fl^*Vil1-Cre* mice, albeit to a lesser extent (Fig. 8b). This prompted us to test whether IFN-γ signaling is activated in the small intestine of *Nfkbiz*^fl/fl^*Vil1-Cre* mice. Indeed, the expression of *Ifng* and *Ciita* was increased (Fig. 8c and Supplementary Fig. 7a), and phosphorylated and total STAT1 were elevated in the intestine (Fig. 8d). Elevated expression of total STAT1 may be explained by an increase in the transcript (Supplementary Fig. 7a).

To examine whether aberrant stimulation with IFN-γ is responsible for decrease in Paneth cells in *Nfkbiz*^fl/fl^*Vil1-Cre* mice, we injected anti-IFN-γ antibody to the mutant mice and analyzed gene expression in the small intestines. The antibody suppressed the expression of IFN-γ-inducible genes and restored the expression of Paneth cell-associated genes, although the effect was moderate (Fig. 8e). Thus, the decrease in Paneth cells in *Nfkbiz*^fl/fl^*Vil1-Cre* mice is attributable to both the enhancement of IFN-γ signaling and impairment of the IL-17-mediated recovery of Paneth cells.

RNA-seq analysis on the jejunum also allowed us to find that expression of the genes encoding IL-1β and TNF-α was increased in the small intestines of *Nfkbiz*^fl/fl^*Vil1-Cre* mice (Supplementary Fig. 7b). Given the role of these cytokines in the potentiation of Th17 differentiation,^50,51^ their higher production is possibly responsible for the increase in Th17 cells in the small intestines of *Nfkbiz*^fl/fl^*Vil1-Cre* mice (Fig. 1b–e). The expression of other genes related to Th17 development, *Il6*, *Il23a*, and *Tgfb* family members, was similar between the small intestines of *Nfkbiz*^fl/fl^*Vil1-Cre* mice and those of control mice (Supplementary Fig. 7c).

Based on the role of IκBζ in chromatin regulation in innate immune responses,^52^ we next investigated genome accessibility in the jejuna of *Nfkbiz*^fl/fl^*Vil1-Cre* mice by assay for transposase-accessible chromatin using sequencing (ATAC-seq). As expected, the accessibility of regulatory regions of *Pigr*, *Cd177*, and *Ccl28* was decreased in the jejuna of *Nfkbiz*^fl/fl^*Vil1-Cre* mice compared to control mice (Supplementary Fig. 8a). On the other hand, the accessibility at Paneth cell-associated genes was only modestly affected by the lack of IκBζ (Supplementary Fig. 8b). These findings are consistent with the notion that Paneth cell integrity is extrinsically regulated by IκBζ in the small intestine (Supplementary Fig. 4a, b), although it is also possible that these Paneth cell-associated genes are not critically regulated by chromatin structure. The accessibility at IFN-γ-inducible genes was increased in *Nfkbiz*^fl/fl^*Vil1-Cre* mice compared to control mice (Supplementary Fig. 8c), which likely contributes to the upregulation of their expression.

### NF-κB p50 is required for IκBζ-mediated gene regulation in IECs

IκBζ preferentially interacts with the NF-κB p50 subunit (encoded by *Nfkb1*) rather than other NF-κB subunits due to the structural features of the ankyrin repeats of IκBζ and the Rel homology domain of p50.^21^ In LPS-stimulated macrophages, p50 was required for expression of the IκBζ-dependent genes *Lcn2* and *Il6* (Supplementary Fig. 9a) and association of IκBζ with the target promoter followed by subsequent recruitment of the NF-κB p65 subunit (Supplementary Fig. 9b).^52^

**Fig. 9:**
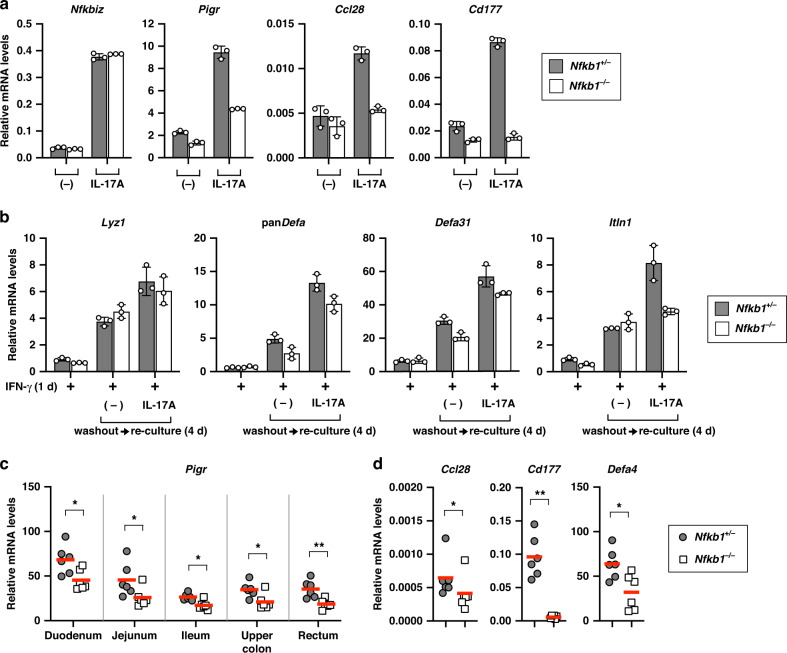
NF-κB p50 is required for IκBζ-mediated gene regulation in IECs. **a** The small intestinal organoids from control (*Nfkb1*^+/−^) or NF-κB p50-deficient (*Nfkb1*^−/−^) mice were unstimulated or stimulated with IL-17A (20 ng/ml) for 24 h. Expression of the indicated genes was analyzed by RT-qPCR. The results are presented as the mean ± SD of triplicates and are representative of organoids from three mice. **b** Organoids from the indicated mice were treated with IFN-γ (20 ng/ml), washed out, and re-cultured in fresh media for 4 days in the absence or presence of IL-17A (20 ng/ml). Expression of the indicated genes was analyzed as in (**a**)**. c**, **d** Total RNA was extracted from the indicated intestinal regions (**c**) or the ileum (**d**) of the indicated mice. Expression of the indicated genes was analyzed by RT-qPCR. The mean expression levels are shown (*n* = 6 mice per group). Statistical significance was determined by Mann–Whitney *U* test (**c**, **d**). *p < 0.05, **p < 0.01, ****p < 0.0001, n.s., not significant.

To elucidate the role of p50 in IκBζ-mediated gene regulation in IECs, we prepared intestinal organoids from *Nfkb1*^–/–^ mice and analyzed the expression of IκBζ-dependent genes (Fig. 9a). Lack of p50 did not impair the expression of *Nfkbiz* (Fig. 9a). IL-17A-induced expression of *Pigr*, *Ccl28*, and *Cd177* was abrogated in p50-deficient organoids (Fig. 9a), whereas the expression of Paneth cell-associated genes was not decreased (Supplementary Fig. 9c) as in the case of IκBζ deficiency (Fig. 6b). In addition, IL-17A less efficiently facilitated the recovery of Paneth cells in p50-deficient organoids than control organoids (Fig. 9b). Consistent with these in vitro findings, expression of *Pigr*, *Ccl28*, *Cd177*, and *Defa4* was decreased in the small intestines of *Nfkb1*^–/–^ mice compared to controls (*Nfkb1*^+/–^) (Fig. 9c, d). Therefore, IκBζ appears to cooperate with p50 in IL-17-mediated regulation of the microbe-controlling genes in IECs.

## Discussion

In the present study, we generated IEC-specific IκBζ-deficient mice (*Nfkbiz*^fl/fl^*Vil1-Cre* mice) and observed an expansion of SFB in the small intestines, thereby promoting Th17 cell development and exacerbating inflammation. The dysbiosis was associated with an impaired expression of a group of genes involved in the control of enterobacteria in IECs. In intestinal organoids, expression of some genes, including *Pigr* and *Ccl28*, was upregulated in an IκBζ-dependent manner following IL-17A stimulation. Although the expression of multiple Paneth cell-associated genes was decreased due to severe impairment of Paneth cells in *Nfkbiz*^fl/fl^*Vil1-Cre* mice, Paneth cells developed normally in the organoids cultured under standard conditions. Importantly, Paneth cells recovered after IFN-γ-induced damage, and the recovery was facilitated by IL-17 in an IκBζ-dependent manner. The decrease in Paneth cells in *Nfkbiz*^fl/fl^*Vil1-Cre* mice was likely caused by enhanced IFN-γ signaling and a defect in the recovery. We also showed that IECs lacking the NF-κB p50 subunit exhibited similar defects, suggesting cooperation between IκBζ and p50 in the IL-17-mediated control of the gut microbiota by IECs.

In addition to its primary role in pro-inflammatory properties, IL-17 also contributes to the maintenance of gut homeostasis by serving multiple functions, including the regulation of Paneth cells.^9–11^ The lack of IL-17 signaling has been shown to cause Paneth cell deficiency, as observed in mice deficient in IL-17 receptor^53^ or RORγt,^54^ but it was unclear how IL-17 signaling regulates Paneth cells. One reason for the unclarity is that Paneth cell functions are not affected by IL-17 under standard in vitro culture conditions. In the present study, we observed the impact of IL-17 on Paneth cells in organoid culture by analyzing the recovery process after the IFN-γ-induced damage. The treatment of the organoids with IFN-γ induces reduction in several types of IECs, but IL-17A selectively enhanced the regeneration of Paneth cells. As the IL-17-mediated recovery of Paneth cells depends on IκBζ, the decrease in Paneth cells in the intestines of *Nfkbiz*^fl/fl^*Vil1-Cre* mice may be at least partly explained by a defect in their recovery. Given that mice harboring IEC-specific deletion of *Rela* (the NF-κB p65 subunit) or *Ikbkg* (the NF-κB signaling molecule NEMO) exhibit similar reduction in Paneth cell numbers,^55^ the NF-κB signaling pathway appears to prevent the defect in Paneth cells through the induction of IκBζ expression in response to IL-17. In addition to the defect in IL-17-mediated recovery of Paneth cells, the decrease in Paneth cells in the intestine of *Nfkbiz*^fl/fl^*Vil1-Cre* mice may also be explained by the enhanced stimulation by IFN-γ.

Despite the essential role of IκBζ in the maintenance of Paneth cell homeostasis, it is unlikely that IκBζ plays the role in Paneth cells under steady state conditions, where Paneth cells do not express IκBζ. While IκBζ is expressed following IL-17A stimulation in Paneth cells recovering from IFN-γ-induced damage, the expression is less than that in Lgr5^+^ IEC stem cells and enterocytes. Recently it was reported that IL-17R signaling in Lgr5^+^ stem cells promotes the development of secretory IEC lineages including Paneth cells.^49^ The process in stem cells is possibly mediated by IκBζ. It is plausible that stem cells and enterocytes release a soluble factor(s) required for Paneth cell maintenance in an IκBζ-dependent manner. As soluble factors that promote the development of Paneth cells, Wnt ligands for Frizzled-5 (Wnt3, Wnt6, and Wnt9) and colony stimulating factor-1 have been reported.^56,57^

The expansion of SFB in *Nfkbiz*^fl/fl^*Vil1-Cre* mice is likely caused by the decrease in IgA production and reduction in Paneth cell-derived anti-microbial proteins. The amount of SFB is increased in the intestine of mice deficient in activation-induced cytidine deaminase, in which hyper-mutated IgA is absent but defensins are normally expressed.^58^ The role of α-defensins in the control of SFB colonization was demonstrated by complementary mouse models: the overexpression of human *DEFA5* decreased SFB in the intestine, while the inference with activation of α-defensins by deletion of the common processing enzyme MMP7 caused increase in SFB.^59^ In contrast to the limited colonization in the ileum of wild-type mice,^43^ SFB were aberrantly extended to the upper regions of the small intestine of *Nfkbiz*^fl/fl^*Vil1-Cre* mice. Based on a recent single-cell RNA-seq analysis showing that Paneth cells are distinct between the upper region and the ileum,^60^ it is possible that anti-microbial factors from the “upper” Paneth cells exclude SFB in wild-type mice, and defects in this function allows SFB to colonize in the upper regions of the intestine of *Nfkbiz*^fl/fl^*Vil1-Cre* mice. Although it is also possible that SFB requires the ileum-specific nutrients for their colonization,^61^ the expression of the region-specific metabolic enzymes was not altered in our transcriptome analysis of the intestine of *Nfkbiz*^fl/fl^*Vil1-Cre* mice.

Th17 cells and SFB constitute a regulatory loop in which Th17 cells interfere with SFB proliferation while SFB upregulate the development of Th17 cells. This regulatory loop may be important for maintaining a balance of SFB and Th17 to optimize host defenses against infection and minimizing risk for autoimmunity. Indeed, elimination of Gram-positive bacteria, including SFB, by administering vancomycin leads to an impairment of anti-fungal activity by Th17 cells in the respiratory tract.^62^ In this regulatory loop, the IL-17–IκBζ signaling axis plays a key role in the inhibition of SFB by Th17 cells, as a lack of IκBζ in IECs results in the expansion of SFB despite an increase in Th17 cells. The increase of IFN-γ^+^ Th17 cells may further reduce the ability to limit SFB by inducing decrease in Paneth cells in *Nfkbiz*^fl/fl^*Vil1-Cre* mice.

Blocking the IL-17–IκBζ axis effectively suppresses the pathology of psoriasis by downregulating the production of inflammatory mediators in the skin.^63^ On the other hand, IL-17–IκBζ signaling in IECs does not appear to induce the production of pro-inflammatory molecules, because the expression of inflammatory mediators was not downregulated in our transcriptome analysis of the intestine of *Nfkbiz*^fl/fl^*Vil1-Cre* mice. Therefore, it is possible that anti-inflammatory effects will not occur with blocking the IL-17–IκBζ axis in IECs, which may instead aggravate inflammation or increases the risk of infections due to dysregulation of the microbiota. The present study also shows that dysbiosis leads to decrease in Paneth cells by inducing the development of IFN-γ-producing cells. Given that IFN-γ-induced decrease in Paneth cells further drives this vicious cycle, the dominance of IL-17 signaling over IFN-γ may be crucial for maintaining intestinal homeostasis and relieving enteritis associated with Paneth cell abnormalities.

## Methods

### Reagents

The following reagents were obtained as indicated: recombinant mouse IL-17A protein (BioLegend 576002), recombinant IFN-γ protein (PeproTech 315-05), lipopolysaccharide (List Biological Laboratories, *Escherichia coli* O111:B4), phorbol 12-myristate 13-acetate (PMA) (SIGMA P8139), ionomycin (I1957), and Golgi Plug (BD Bioscience, 555029).

### Mice

A targeting vector for the *Nfkbiz*^fl/fl^ mice was constructed to delete exons 5, 6, and 7 of the *Nfkbiz* gene, which encode ankyrin repeats of IκBζ (Supplementary Fig. 1c). Genomic fragments of *Nfkbiz* were generated by PCR and cloned with loxP sequences and a *Neo*^r^ cassette flanked by FRT sequences as shown in Supplementary Fig. 1c. For the combinatorial use of CRISPR/Cas (clustered regularly interspaced short palindromic repeats/CRISPR associated proteins) system, three sets of sgRNA oligos (5′-caccgaaggggtgcgggaacagtc-3′ and 5′-aaacgactgttcccgcaccccttc-3′ for pX335-G1, 5′-caccgagatagctgtctgagtacgc-3′ and 5′-aaacgcgtactcagacagctatctc-3′ for pX335-G2 and 5′-caccgtatcaatgtatcgttaaat-3′ and 5′-aaacatttaacgatacattgatac-3′ for pX-335-G3) were cloned into BbsI-digested pX335-U6-Chimeric_BB-CBh-hSpCas9n(D10A) plasmid (Addgene #42335). C57BL/6N-derived ES cell line, 6NK7 ES cells^64^ (2 × 10^6^ cells) were co-electroporated with circular forms of 20 μg of the targeting vector and 10 μg each of pX335-G1, 2 and 3 using Gene Pulser Xcell^TM^ (Bio-Rad) and plated onto two 10 cm plates. After neomycin selection, ES clones in which single transgene was integrated into the *Nfkbiz* locus, were obtained. ES cells were aggregated with ICR morula as described.^65^ The chimeric mice were mated with C57BL/6 mice to establish a strain with a germline-transmitted locus. The resultant mice were bred with FLPe-expressing mice^66^ to delete *Neo*^r^ cassette flanked by FRT sequences. FLPe-expressing mice^66^ were obtained from Riken BioResource Research Center (RBRC01834, C57BL/6-Tg(CAG-flpe)36Ito/ItoRbrc). *Vil1-Cre* mice,^32^ *Lyz2*^Cre^ mice,^67^ and *Nfkb1*^–/–^ mice^68^ were purchased from The Jackson Laboratory. *Lyz2*^Cre^ mice were used for gene deletion in Paneth cells, as *Lyz2* is specifically expressed in Paneth cells among IECs.^60,69^ All mice were maintained under SPF conditions in the animal facility at Toho University School of Medicine. The experimental protocols were approved by the Toho University Administrative Panel for Animal Care (22-54-413) and Recombinant DNA (22-54-410 and 22-53-442). Every effort was made to minimize the number and suffering of mice.

### Fractionation of the small intestinal tissue

The terminal ilea isolated from wild-type mice (~10 cm) were incised longitudinally and the luminal contents were washed out in PBS. The tissue was treated with 10 mM EDTA in RPMI medium for 30 min at 37 °C. The dissociated cells were used as epithelial fraction after filtration with a 70 μm cell strainer (greiner bio-one 542070). The remaining tissue was washed in RPMI with vigorous shaking, and the middle portion (~1 cm) was used as lamina propria.

### Animal disease models

In all disease model experiments, we used gender- and age-matched mice that had been co-housed since their birth. For DSS-induced colitis model, the disease was induced in mice by oral administration of 2.0% DSS (MW 36,000–50,000, MP Biomedicals Inc) ad libitum in drinking water for 5 days, and then normal drinking water was given in the following days. For induction of enteritis in the small intestine, an anti-CD3ε agonistic antibody (self-made, clone 145-2C11) was peritoneally injected to mice at day 0, 2, and 4 (1.0 mg/kg). In these two intestinal inflammation models, body weight was measured every 24 h, and the relative values to the initial body weight was shown. For induction of EAE model, mice were subcutaneously injected with the synthetic antigen peptide MOG_35–55_ (100 μg per mouse, Scrum Inc.) emulsified in complete Freund’s adjuvant without additional Mycobacterium tuberculosis H37RA. Pertussis toxin (500 ng per mouse, Calbiochem) was intraperitoneally injected to the mice on days 0 and 2. The severity of the disease was scored as follows: 0, no clinical signs; 1, tail limpness; 2, hind limb weakness; 3, hind limb paralysis; 4, fore limb weakness; 5, quadriplegia; 6, death.

### In situ hybridization

Mice were perfused transcardially with 4% (w/v) paraformaldehyde (PFA) in phosphate-buffered saline (PBS, pH 7.4). The guts were removed, postfixed in 4% PFA at 4 °C overnight, followed by cryoprotection in 30% (w/v) sucrose for a day. The whole small intestine was cut longitudinally and the “Swiss roll” was made. The rolled tissue was embedded in the Surgipath (FSC22, Leica Biosystems), cryo-sectioned at the thickness of 20 μm, and then stored at −20 °C until use. Multicolour fluorescent in situ hybridization chain reaction was performed as previously described^33^ with some modifications using reagents from Nepagene Co. Ltd. (Chiba, Japan). Briefly, the section was treated with methanol for 10 min at room temperature, and then incubated in the hybridization solution containing 5× SSC, 10% dextran sulfate (MW. 500,000; Wako), 30% formamide, 0.1% Tween-20, 1× Denhardt’s solution and 50 μg/ml heparin for more than 5 min at 37 °C. After denaturing for 5 min at 95 °C, the DNA probe was diluted to the concentration of 10 nM with the hybridization solution, and applied onto the section. The section was covered by a piece of parafilm sheet, and incubated at 37 °C overnight in a moist chamber. After the section was sequentially washed with the solution containing 5× SSC, 30% formamide, and 0.1% Tween-20 for 10 min, followed by 5× SSC (10 min), the autofluorescence was quenched for an hour by the LED illuminator (TiYO, Nepagene, Japan). The section was incubated with the amplification buffer containing 8× SSC, 10% dextran sulfate, 0.2% Triton X-100, and 100 mM MgCl_2_ for more than 5 min, and then with the fluorophore-conjugated hairpin DNA (each 60 nM) for 2 h at 25 °C. Hairpin DNAs were snap-cooled (heated at 95 °C for 1 min, slowly cooled to 65 °C for 15 min, and then 25°C for 40 min) to form a hairpin structure before use. Finally, the section was stained with Hoechst 332342 (1 μg/ml) and mounted with anti-fade reagent (VECTASHIELD Mounting Medium, Vector Laboratories). The stained section was examined under a confocal laser microscope (A1R; Nikon, Tokyo, Japan). The nucleotide sequences of specific probes and hairpin DNAs were shown in Supplementary Table 1 and Supplementary Table 2, respectively.

In situ hybridization analysis for small intestinal organoids was conducted by RNAscope (Advanced Cell Diagnostics). After cultured on a chamber slide (Matsunami Glass, SCS-N08), organoids were fixed with formaldehyde (10%) and analyzed according to the manufacture’s protocol (https://www.cosmobio.co.jp/support/technology/document/ADC_Tech_Note_Mux_FL_CulturedCells_V2.pdf) using specific probe to *Nfkbiz* (806551), *Lyz1* (415131-C2), *Enpep* (862211-C3), and *Lgr5* (312171-C3). Pictures were obtained using the BZ-X700 All-in-one microscope (KEYENCE) or the confocal laser microscope LSM880 (Zeiss). Images were analyzed using BZ-X Analyzer (KEYENCE) or ZEN software (Zeiss).

### Analysis on bacterial flora

DNA was isolated from the intestinal tissues and feces, and microbiota analysis was carried out as previously described.^44^ Briefly, the V4 region of the 16 S rRNA gene was amplified by PCR using barcoded dual-index primers that contain an Illumina adaptor and the V4-specific primers F515 and R806.^70^ The amplified fragments were pooled into a library, and the both ends of the fragments were sequenced using an Illumina MiSeq instrument. The obtained sequences were curated using Mothur (v.1.40.5), in which the sequences were binned into OTUs at >97% identity level and taxonomically assigned. After calculation of relative abundancies of individual OTUs and higher taxons, the indexes for α-diversity and β-diversity were obtained. Shannon evenness, which provides information on how much equal the abundancies of the OTUs are in a microbiome, was obtained by dividing Shannon index by natural logarithm of the total number of OTUs.

### Measurement of IgA amount

The amount of IgA in feces was measured by enzyme-linked immunosorbent assay (ELISA) using Mouse IgA ELISA Kit (Bethyl laboratories, inc E99-103). The fecal IgA content was determined after normalization with the amount of total fecal protein measured using Protein Assay Dye Reagent (Bio-Rad 5000006).

### Quantitative polymerase chain reaction (qPCR)

Quantitative PCR (qPCR) was conducted using FAST SYBR^TM^ Green Master Mix (Applied biosystems, 4385614) on QuantStudio 3 real-time PCR system (Applied biosystems). The sequences of qPCR primers used were listed in Supplementary Table 3.

### Gene expression analysis by reverse transcription–qPCR (RT-qPCR)

Total RNA was isolated using Sepasol^Ⓡ^-RNA Super G (nacalai tesque). For extraction of RNA from the intestinal tissues, the tissue fragment was ground with a Zirconia bead (TOMY, ZB-50) in Sepasol^Ⓡ^-RNA Super G using Micro Smash MS-100R (TOMY Digital Biology). The obtained RNA was reverse-transcribed to complementary DNA (cDNA) using ReverTra Ace^Ⓡ^ qPCR RT kit (TOYOBO, FSQ-101). cDNA was used as a template in qPCR. The relative expression level of every gene was determined after normalization to that of the house-keeping gene *Hprt*.

### Measurement of the bacteria contents in the intestinal tissues and feces

DNA was extracted from mouse intestinal tissues and feces using QIAamp DNA Mini Kit (QIAGEN, 51304) and QIAamp Fast DNA Stool Mini Kit (QIAGEN, 51604), respectively. The obtained DNA was used as a template of qPCR. The content of bacteria was determined after normalization to the amount of total eubacteria or *Actb*.

### Chromatin immunoprecipitation (ChIP)

ChIP assay was essentially performed as previously described.^52^ Cells were fixed with formaldehyde (1.0%) for 10 min at room temperature, and sonicated. The fragmented chromatin was subjected to immunoprecipitation using anti-IκBζ antibody,^31^ anti-NF-κB p65 antibody (Santa Cruz, sc-372), or rabbit control IgG (GeneTex, GTX35035). After reversal of the cross-linking, the precipitated DNA was analyzed by qPCR. The association of the transcription regulator with the target site was given after normalization to the amount of input DNA.

### Immunoblotting

Immunoblotting was performed according to the standard protocol using antibodies against total-STAT1 (Cell signaling Technologies 9172), phospho-STAT1 (S727) (Cell signaling 8826S), and α-tubulin (Sigma-Aldrich T5168).

### Microarray chip analysis

Total RNA was prepared from the ilea of two pairs of *Nfkbiz*^fl/fl^*Vil1-Cre* mice and co-housed gender-matched control (*Nfkbiz*^fl/fl^) mice. Labeling of cRNA was performed using Agilent Low Input Quick Amp Labeling Kit following the manufacture’s instructions (Agilent Technologies). Briefly, after total RNA was reverse-transcribed to double-stranded cDNA using a poly(dT)-T7 promoter primer, the resultant cDNA was used as a template for in vitro transcription in the presence of Cyanine 3 (Cy3)-CTP. The Cy3-labeled cRNA was fragmented, and hybridized onto Agilent SurePrint G3 Mouse GE v3 8 × 60 K Microarray (Design ID: 074809). After washed, the microarray was scanned using an Agilent SureScan Microarray Scanner (G4900DA). Intensity values of each scanned feature were quantified using Agilent feature extraction software version 12.1.1.1, which performs background subtractions. We employed features that were fragged as no errors (Detected flags), and excluded features that were not positive, not significant, not uniform, not above background, saturated, and population outliers (Not Detected and Compromised flags). Quantile normalization was performed using Agilent GeneSpring software 14.9.1.

### Flow cytometric analysis on the lamina propria lymphocytes in the small intestine

Non-epithelial cells were prepared from the lamina propria of the small intestine as described previously.^71^ After epithelium was removed by incubation in 2 mM EDTA, the remaining intestinal tissue was chopped into small pieces, and digested with collagenase (0.5 mg/ml, Wako). The obtained cell suspension was suspended in 40% Percoll solution (GE Healthcare) and loaded on a 80% Percoll solution. After centrifugation for 20 min at 880 × *g* at room temperature, cells at interface between the two Percoll solutions were harvested. After stimulation for 4 h with PMA (50 ng/ml) and ionomycin (500 ng/ml) in the presence of GolgiPlug (1:1000 dilution), cell surface was stained with FVD506 (eBioscience 65-0866-14), APC/Cy7-labeled anti-CD45.2 antibody (BioLegend 109824, clone 104), APC-labeled anti-CD4 antibody (TONBO 20-0042, clone RM4-5), PE/Cy7-labeled TCRβ antibody (TONBO 60-5961, clone H57-597). The cells were fixed with Intracellular Fixation Buffer (eBioscience 00-8222) and then perforated in Permeabilization Buffer (eBioscience 00-8333). Intracellular cytokines were stained with PE-labeled anti-IL-17A (BioLegend 506904, clone TC11-18H10) and FITC-labeled anti-IFN-γ (BioLegend 505806, clone XMG1.2) in Permeabilization Buffer, and analyzed using LSR Fortessa^TM^ X-20 Cell Analyzer (BD Bioscience) and FlowJo (BD Bioscience).

### Preparation of the small intestinal organoid culture

Preparation and maintenance of the small intestinal organoid culture were carried out according to the instruction of Intestinal Epithelial Organoid Culture with IntestiCult^TM^ Organoid Growth Medium (Mouse) (STEM Cell technologies 06005). For dissociation of crypts from the intestinal tissue, the jejunum was cut into small fragments, extensively washed with cold PBS, and incubated at 4 °C in 10 mM EDTA. The isolated crypts were suspended in Matrigel (Corning 356231) and cultured in the complete medium (STEM Cell technologies 06005). For inoculation, organoid-containing Matrigel was dissociated with Gentle Cell Dissociation Reagent (STEM Cell technologies 07174), and the organoids were washed with DMEM/F-12 with 15 mM HEPES (STEM Cell technologies 36254), and re-suspended in Matrigel for re-plating. For the experiments of recovery from IFN-γ-induced Paneth cell death, the IFN-γ-treated organoids were washed twice, and re-cultured in fresh media under IFN-γ-free conditions

### Preparation of bone marrow-derived macrophages

Bone marrow-derived macrophages were prepared as described previously,^52^ and maintained in Dulbecco’s modified Eagle’s medium supplemented with 10% heat-inactivated fetal calf serum, penicillin (100 units/ml), and streptomycin (100 μg/ml).

### Histological analysis

The tissue from the intestine or the spinal cord was fixed in formaldehyde (10%) overnight at room temperature, and embedded in paraffin blocks. The paraffin-embedded section was used for H&E staining or immunohistochemical analysis. For immunohistochemical analysis, paraffin-embedded section was autoclaved in citrate buffer (LSI Medicine, RM-102C) for retrieval of the antigens, and then stained with anti-Lysozyme antibody (abcam, ab108508). After treatment with HRP-conjugated secondary antibody using ImmPRESS VR Polymer HRP Anti-Rabbit IgG Reagent (Vector Laboratories, MP-6401), the distribution of Lysozyme was visualized with 3-3′-diaminobenzidine (nacalai tesque, 11009-41). The section of the spinal cord was stained with Luxol Fast Blue (Muto Pure Chemicals, 4100-1) followed by H&E staining. Small intestinal organoids cultured on a coverslip were fixed with PFA (4%), and permeabilized in a buffer containing normal goat serum (5%) and Triton X-100 (0.5%) in BSA (2%). For fluorescent analyses, sections were stained with fluorescein-labeled *Ulex Europaeus* agglutinin 1 (UEA-1) (Vector laboratories, FL-1061-2), Alexa 647-labeled anti-E-Cadherin antibody (BD, 560062 clone 36/E-Cadherin), and Hoechst 33258 (Nacalai, 04928-92). Pictures for the images were obtained using the BZ-X700 All-in-one microscope (KEYENCE). Images of organoids were processed using acquisition of Z-stacks followed by haze reduction function.

### RNA sequencing (RNA-seq)

The 5′ RNA sequencing was performed by ImmunoGeneTeqs, Inc. (Chiba, Japan). PolyA^+^ RNA was isolated using Dynabeads M-270 Streptavidin (Thermo Fisher Scientific) and biotin-3′ WTA-EcoP-dT25, according to the previous analysis (GSE110711) with some modifications. After reverse transcription and template switching, the cDNA was amplified and subjected to fragmentation/end-repair/polyA-tailing/ligation using NEBNext Ultra II FS DNA Library Prep Kit for Illumina (New England Biolabs). Barcoded libraries (~300 bp) were obtained by PCR using NEBNext Ultra IIQ5 Master Mix (New England Biolabs), and sequenced on the Illumina Novaseq 6000 S4 flowcell (Illumina). After adaptor trimming of sequencing data in single-end fastq files using cutadapt 2.10, the reads were mapped to reference RNA (mRNA and ncRNA of GRCm38 release 101) with bowtie 2-2.3.4.2. The reads in each gene were counted using awk, sort and uniq -c commands. The count data was summarized, and the expression table was full-outer joined by gene symbols using Microsoft R open-3.5.3 and dplyr-1.0.0 package.

### Injection of anti-IFN-γ antibody

Co-housed *Nfkbiz*^fl/fl^*Vil1-Cre* mice were intraperitoneally injected with an isotype control antibody (ichorbio ICH2246) or an anti-IFN-γ antagonistic antibody (clone R4-6A2) (15 mg/kg) every 3 days. Twenty-four hours after the last injection, mice were sacrificed to remove the intestinal tissues.

### Assay for transposase-accessible chromatin using sequencing (ATAC-seq)

The cryopreserved jejunum was sent to Active Motif for ATAC-seq experiments. The nuclei isolated from the tissue were tagmented (fragmented and tagged with sequencing adaptors) by hyperactive Tn5 transposase as described previously^72^ with some modifications^73^ using the reagents in Nextera Library Prep Kit (Illumina). After amplification with ten cycles of PCR, the resultant DNA was sequenced with PE42 sequencing on the NextSeq 500 sequencer (Illumin). Sequence reads were aligned using the BWA algorithm,^74^ and peaks in the histograms were identified using the MACS 2.1.0 algorithm at a cutoff of *p* value 1 × 10^7^. Signal maps and peak locations were analyzed using Active Motifs proprietary analysis program, and reads counted in all merged peak regions were compared using DeSeq2.^75^

### Statistical analysis

For statistical analysis of mouse experiments, pooled data from independent experiments are presented unless otherwise indicated. All statistical analyses were conducted using Prism software (version 9.2.0) and the details of each experiment are shown at the end of the respective figure legend. Significance of the statistics is defined as *p value < 0.05, **p value < 0.01, ***p value < 0.001, ****p value < 0.0001.

## Supplementary information

Author Checklist

Supplementary information

## Data Availability

Raw sequences of microbiome analysis are available via NCBI Short-Read Archive (SRA) with BioProject number PRJNA767592. Raw data of microarray (GSE188196), RNA-seq (GSE188252), and ATAC-seq (GSE188253) are deposited to GEO with reference Series number GSE188254.
