# Supplementary material for: IκBζ controls IL-17-triggered gene expression program in intestinal epithelial cells that restricts colonization of SFB and prevents Th17-associated pathologies
Source: Mucosal Immunol. 2022 Aug 24;15(6):1321–37. doi: 10.1038/s41385-022-00554-3 (PMC9705257; doi:10.1038/s41385-022-00554-3)
Supplement: Supplementary file 2 — Supplementary information [file 41385_2022_554_MOESM2_ESM.pdf]

Supplementary Fig. 1

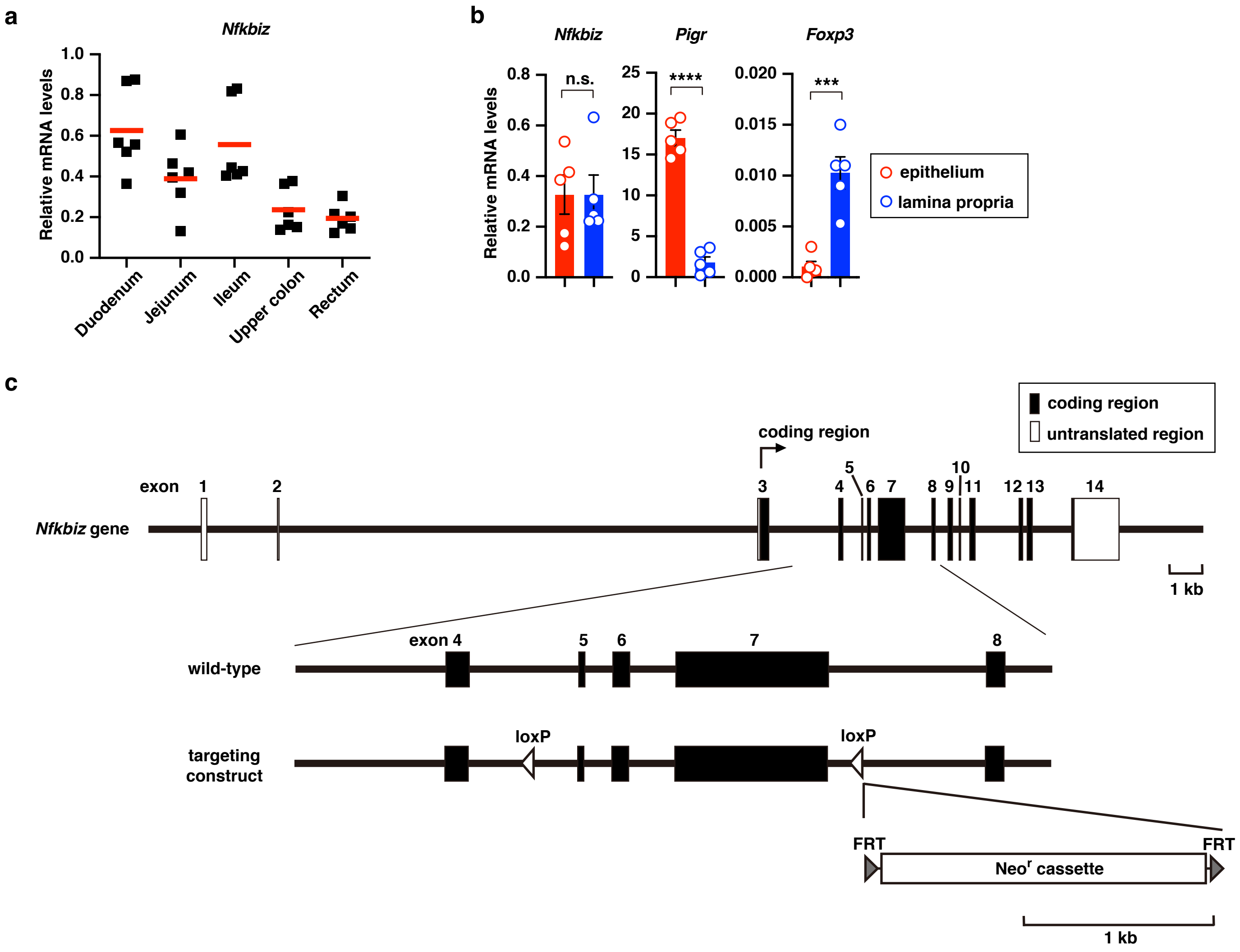

Supplementary Fig. 1 IκBζ is expressed in the epithelium of the small intestine.

a Total RNA was extracted from the indicated gastrointestinal regions of wild-type mice, and mRNA expression level of *Nfkbiz* was determined by RT-qPCR. The mean expression levels are shown (n=6 mice). **b** The ileal tissue prepared from wild-type mice was treated with 10 mM EDTA for 30 min at 37°C. Total RNA was extracted from the dissociated cells (epithelium) or the remaining tissue (lamina propria), and the expression of the indicated genes was analyzed by RT-qPCR. The validity of the fractionation was evaluated by the expression of the marker genes *Pigr* (epithelium) and *Foxp3* (lamina propria). Results are means ±SEMs (n=5). Statistical significance was determined by two-tailed Student's unpaired *t* test. *** $p<0.001$, **** $p<0.0001$, ns, not significant. **c** Targeting strategy of *Nfkbiz* is depicted. Closed and open boxes show protein-coding and untranslated regions, respectively. The targeting construct contains Neo^r cassette flanked by FRT sequences. Mice harboring the targeting construct were crossed to FLP-Tg mice to obtain the floxed *Nfkbiz* ($Nfkbiz^{fl}$) allele by deleting the Neo^r cassette.

Supplementary Fig. 2

a

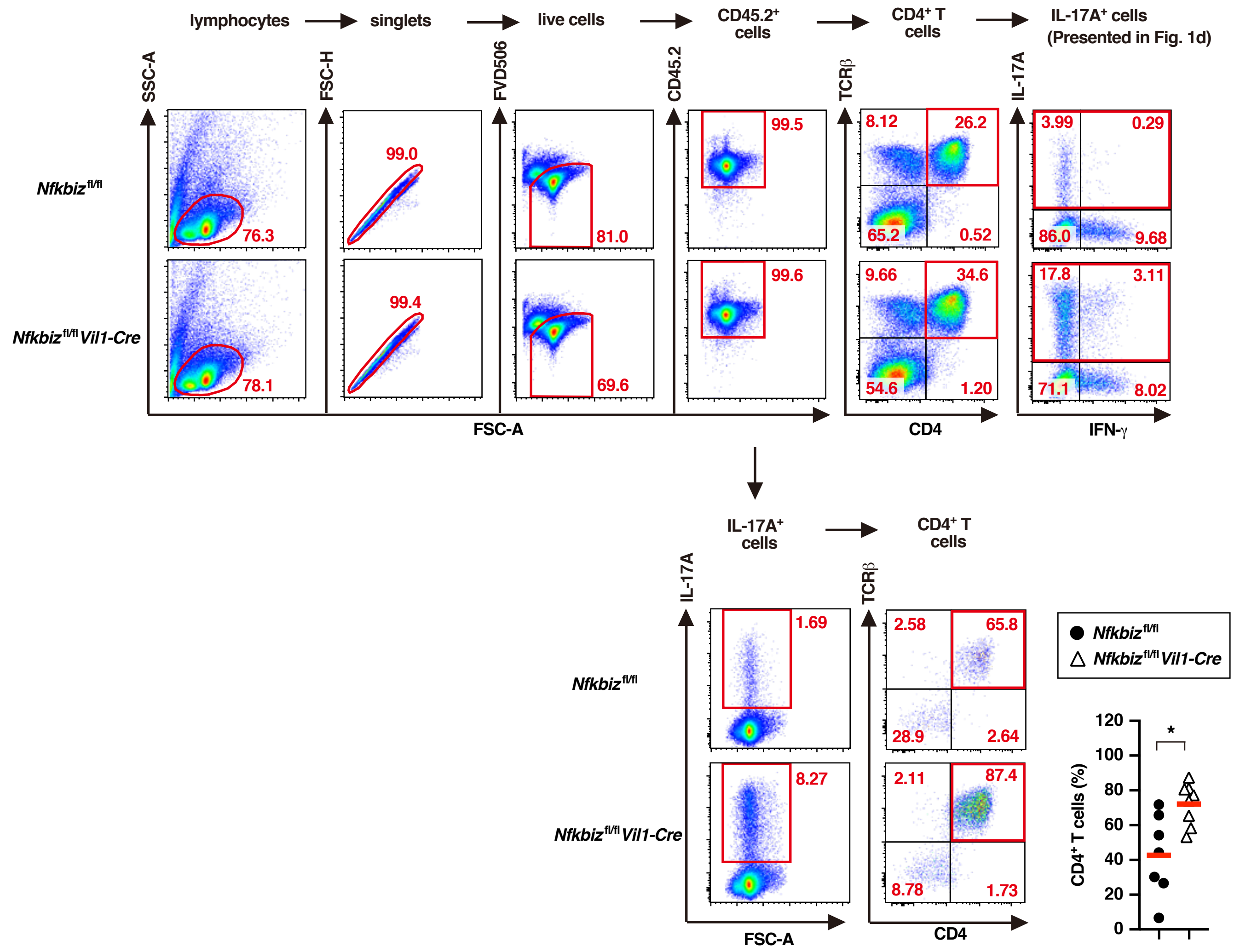

b

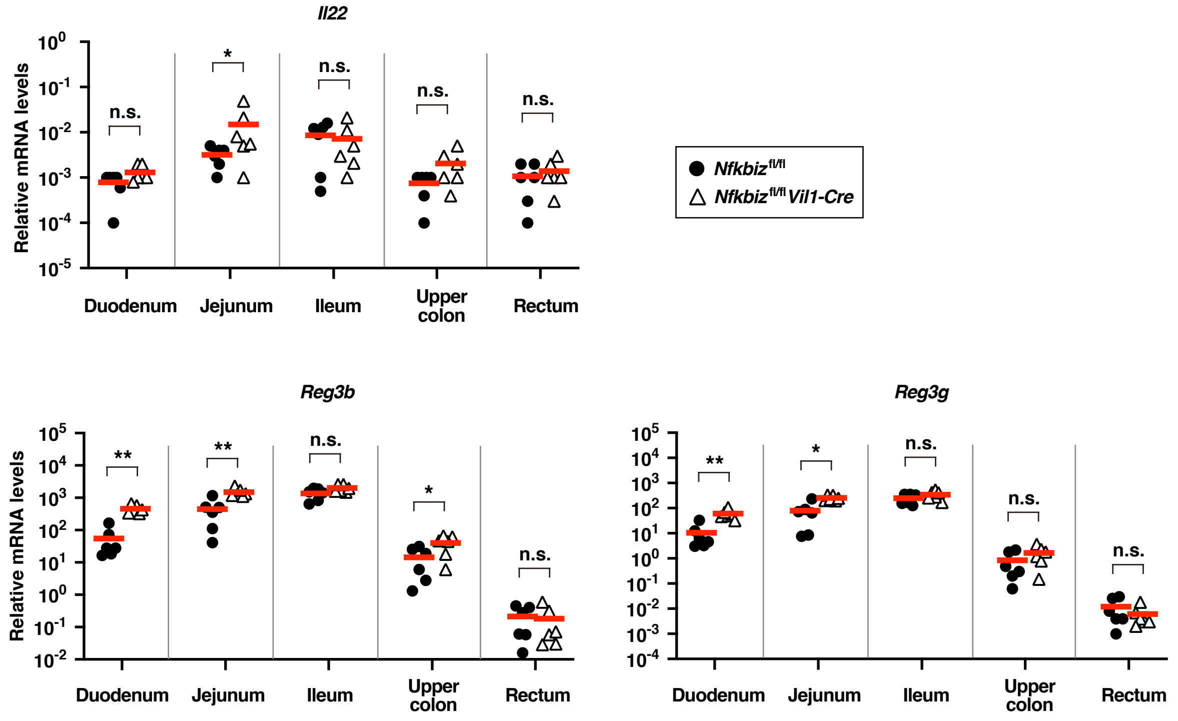

Supplementary Fig. 2 Deletion of IκBζ in IECs causes an aberrant increase in Th17 cells in the small intestine. **a** The gating strategy of flow cytometry analysis in Fig. 1d is shown in the upper panels. The alternative strategy is shown in the bottom. In the right-bottom graph, the percentages of $CD4^{+}TCR\beta^{+}$ cells among $FVD506^{-}$ $CD45^{+}$IL-$17A^{+}$ cells are shown (n=7–8 mice per group). **b** Total RNA was extracted from the indicated gastrointestinal regions of *Nfkbiz*$^{fl/fl}$ *Vil1-Cre* mice or control (*Nfkbiz*$^{fl/fl}$) mice. mRNA expression of the indicated genes was analyzed by RT-qPCR. The mean expression levels are shown (n=6 per group). Statistical significance was determined by Mann–Whitney *U* test. $*p<0.05$, $**p<0.01$, n.s. not significant.

Supplementary Fig. 3

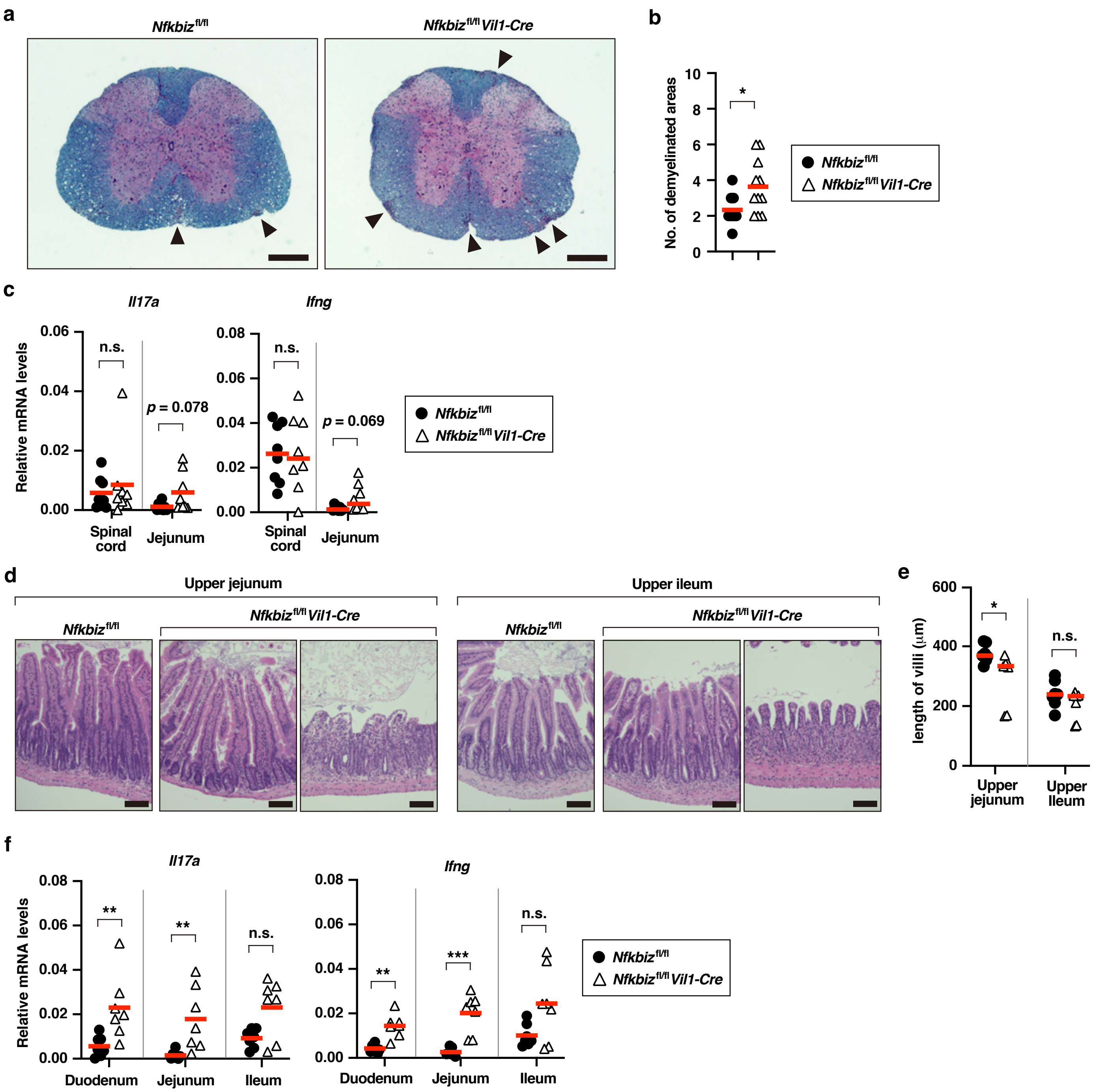

Supplementary Fig. 3 Deletion of IκBζ in IECs causes exacerbation of Th17-mediated inflammatory diseases. **a, b** Sections of the spinal cord were stained with Luxol fast blue and hematoxylin-eosin (H&E). Results are representative of six independent experiments. Demyelinated regions are indicated by arrowheads. Scale bar, 250 μm **(a)**. The mean numbers of demyelinated areas are shown **(b)**. **c** The expression of *Il17a* and *Ifng* was determined by RT-qPCR. The mean expression levels are shown (n=8 mice). **d, e** Sections of the indicated intestinal regions at day 4 were stained with H&E. Results are representative of seven independent experiments. The intestine of *Nfkbiz*$^{fl/fl}$ *Vil1-Cre* mice exhibiting flattened epithelium are shown to the right. Scale bar, 100 μm **(d)**. The lengths of ten villi per mouse were measured, and the mean values are shown (n=7 mice) **(e)**. **f** The expression of *Il17a* and *Ifng* was determined by RT-qPCR. The mean expression levels are shown (n=7 mice). Statistical significance was determined by Mann–Whitney *U* test (**b, c, e, f**). *p<0.05, **p<0.01, ****p<0.0001, n.s., not significant.

Supplementary Fig. 4

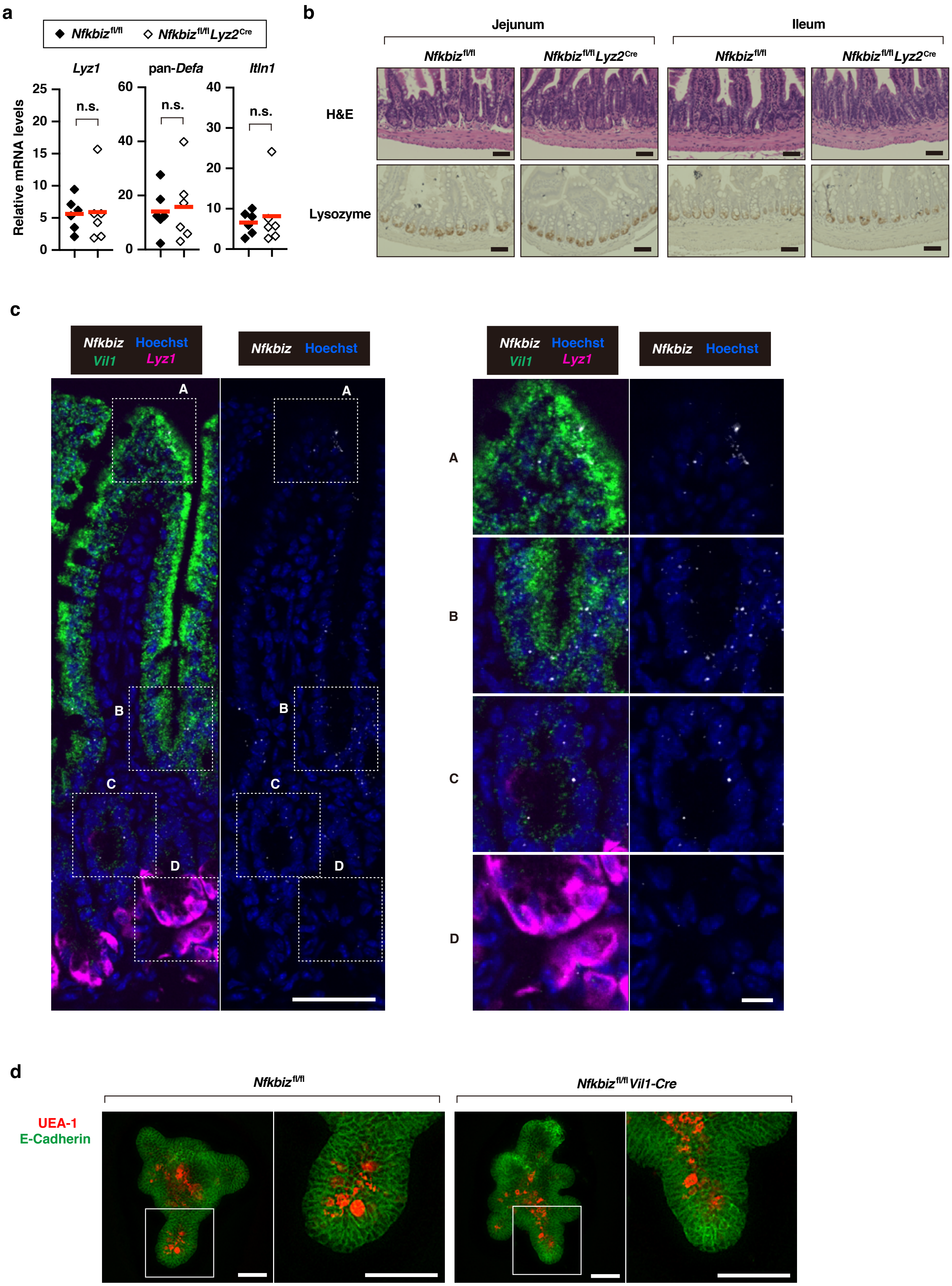

Supplementary Fig. 4 Paneth cells poorly express IκBζ in the small intestine of mice. **a** Total RNA was extracted from the ilea of *Nfkbiz*$^{fl/fl}$*Lyz2*^{Cre} mice or control (*Nfkbiz*$^{fl/fl}$) mice. Expression of the indicated genes was analyzed by RT-qPCR. The mean expression levels are shown (n=6 mice per group). Statistical significance was determined by Mann–Whitney *U* test. n.s., not significant. **b** Tissue section of the indicated small intestinal regions from the control (*Nfkbiz*$^{fl/fl}$) or *Nfkbiz*$^{fl/fl}$*Lyz2*^{Cre} mice was stained with hematoxylin-eosin (H&E) or anti-Lysozyme antibody (n=4 mice per group). **c** A section of the ileum was prepared from control (*Nfkbiz*$^{fl/fl}$) mice and the expression of the indicated genes was analyzed by in situ hybridization. Magnified images of the indicated boxes are shown to the right. Results are representative of three independent experiments. Scale bar, 50 μm (left) and 10 μm (right). **d** Organoids from control (*Nfkbiz*$^{fl/fl}$) or *Nfkbiz*$^{fl/fl}$*Vil1-Cre* mice were stained with UEA-1 and anti-E-cadherin antibody. Results are representative of four independent experiments. Magnified images of the box are shown to the right. Scale bar, 50 μm.

Supplementary Fig. 5

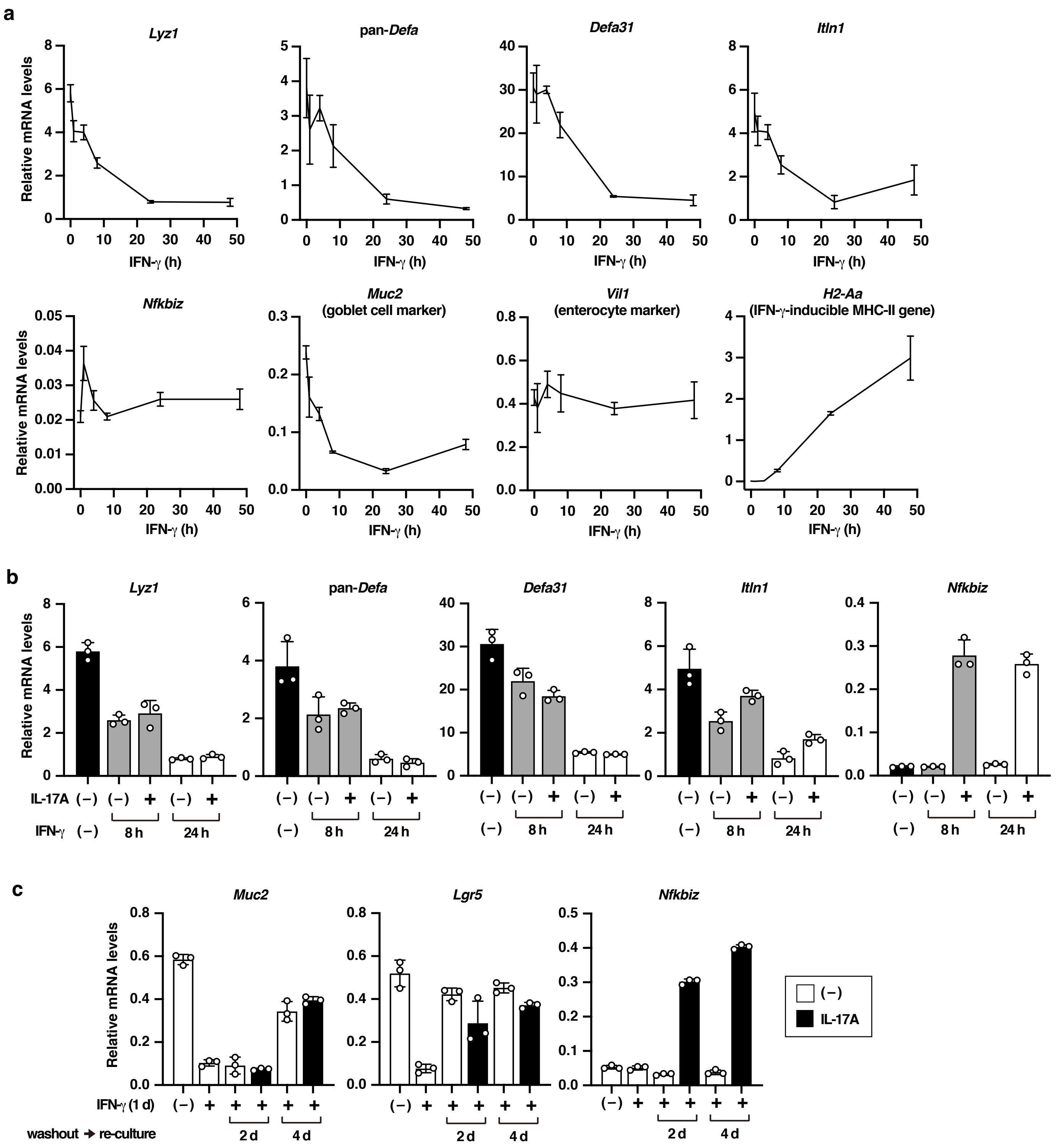

Supplementary Fig. 5 IFN-γ induces decrease of Paneth cells. **a** Small intestinal organoids from wild-type mice were stimulated with IFN-γ (20 ng/ml) for the indicated periods, and expression of the indicated genes was analyzed by RT-qPCR. Results are presented as the mean ±SD of triplicates and are representative of organoids from three mice. **b** Small intestinal organoids from wild-type mice were stimulated with IFN-γ (20 ng/ml) in the absence or presence of IL-17A (20 ng/ml) for the indicated periods. Expression of the indicated genes was analyzed as in (**a**). **c** Small intestinal organoids from wild-type mice were stimulated with IFN-γ (20 ng/ml) for 1 day. Then the organoids were washed twice, and re-cultured in fresh media for 2 or 4 days. Expression of the indicated genes was analyzed as in (**a**).

Supplementary Fig. 6

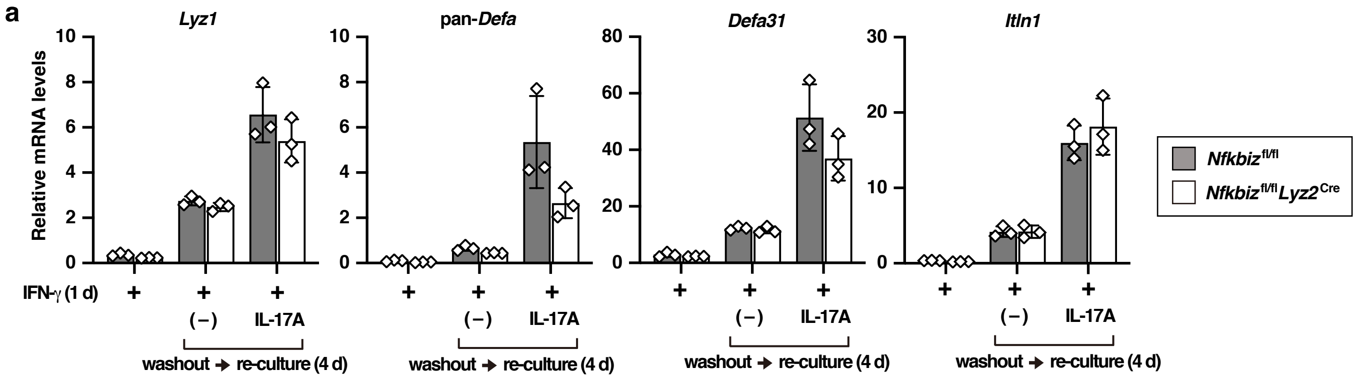

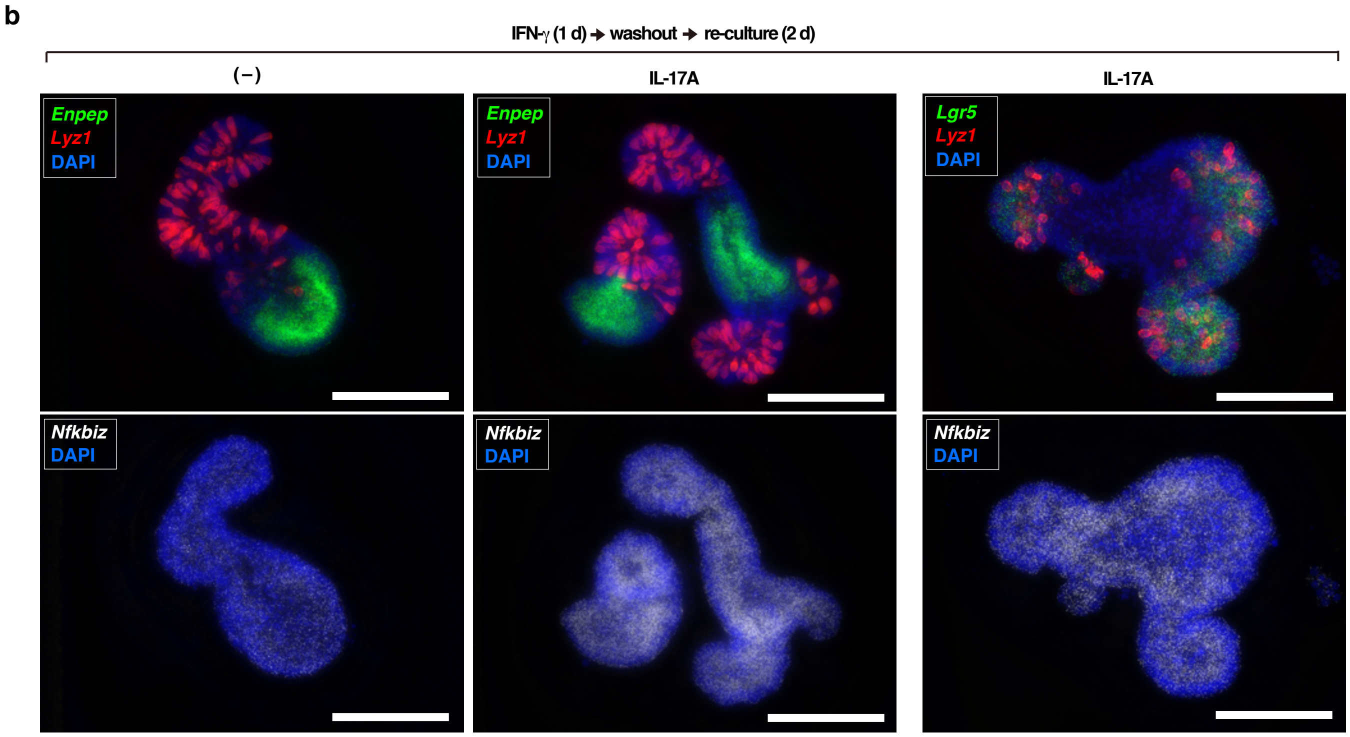

Supplementary Fig. 6 IκBζ is expressed in response to IL-17 in recovering Paneth cells after IFN-γ-induced damage. **a** Organoids from control (*Nfkbiz*$^{fl/fl}$) or *Nfkbiz*$^{fl/fl}$*Lyz2*^{Cre} mice were treated with IFN-γ (20 ng/ml) for 1 day. The organoids were washed out, and re-cultured for 4 days in the absence or presence of IL-17A (20 ng/ml). Expression of the indicated genes was analyzed by RT-qPCR. Results are presented as the mean ±SD of triplicates and are representative of organoids from three mice. **b** Wild-type organoids treated as indicated were analyzed by *in situ* hybridization (RNAscope) using probes specific to *Nfkbiz*, *Enpep* (an enterocyte marker), *Lyz1* (a Paneth cell marker), and *Lgr5* (an intestinal stem cell marker). Results are representative of two independent experiments. Scale bar, 100 μm. Note that the expression of *Enpep* was excluded from the crypts, while that of *Lgr5* was distributed to the crypts.

Supplementary Fig. 7

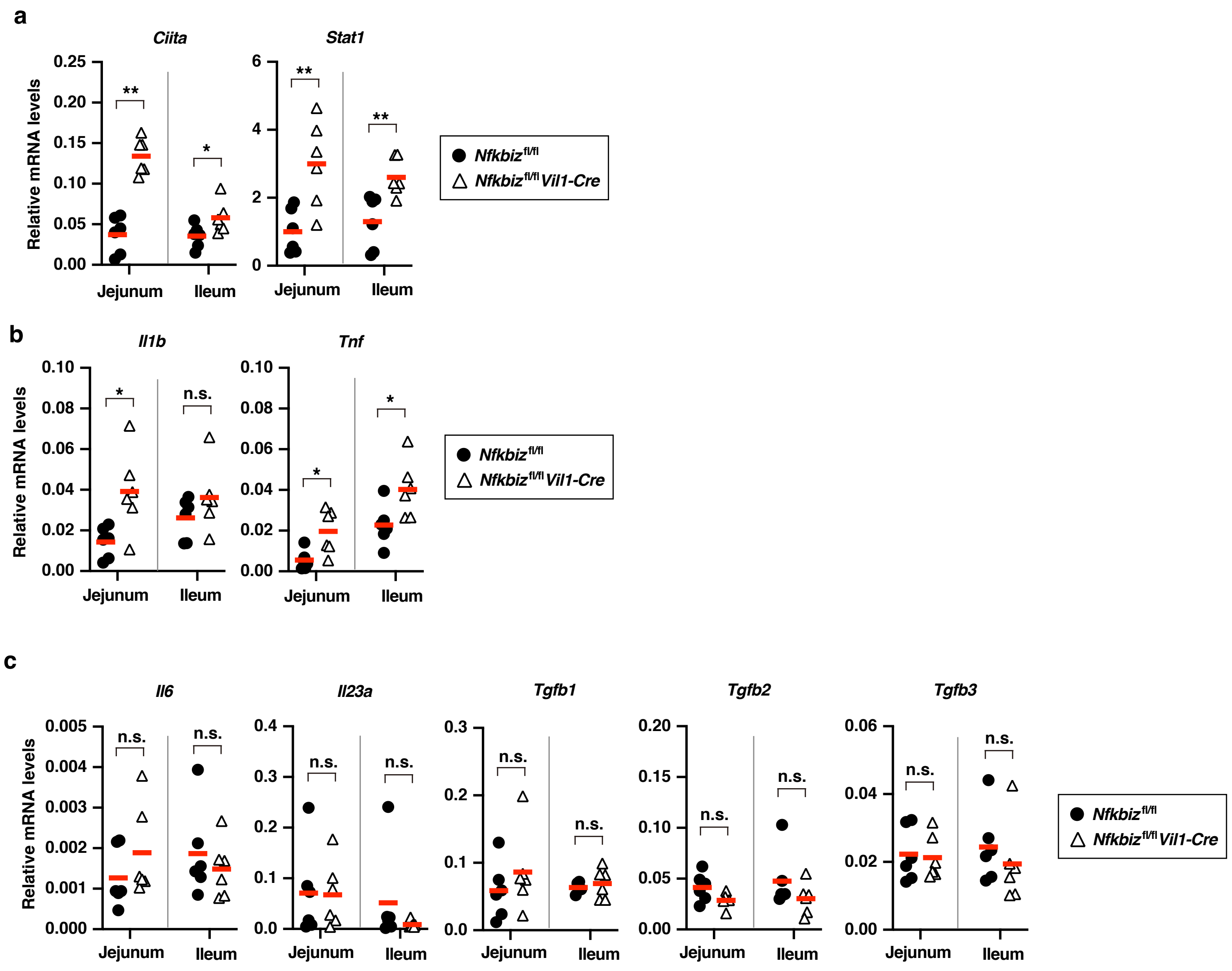

Supplementary Fig. 7 Lack of IκBζ in IECs causes up-regulation of genes involved in IFN-γ signaling and potentiation of Th17 cell development. a–c Total RNA was extracted from the jejuna or the ilea, and the expression of the indicated genes was analyzed by RT-qPCR. The mean expression levels are shown (n=6 mice per group). Statistical significance was determined by Mann–Whitney *U* test. * $p<0.05$, ** $p<0.01$, n.s. not significant.

Supplementary Fig. 8

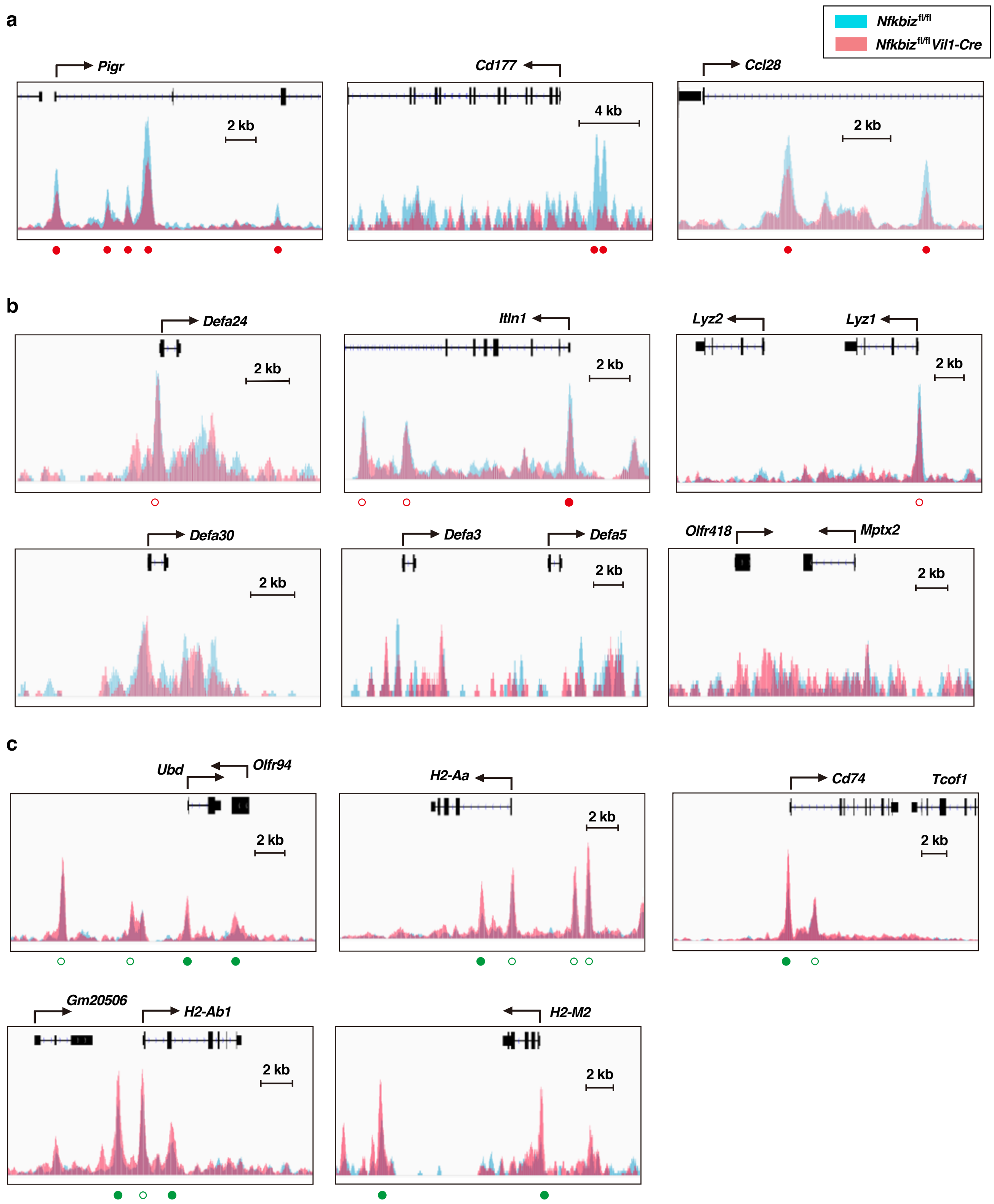

Supplementary Fig. 8 IκBζ regulates accessibility of specific genome regions in IECs of the small intestine. **a–c** ATAC-seq analysis was conducted using the jejuna of the indicated mice. The opened chromatin regions at IL-17-iducible genes (**a**), Paneth cell-associated genes (**b**), and IFN-γ-inducible genes (**c**) are shown in peaks. Red and green closed dots indicate peaks <75% and >150% change in the jejuna of *Nfkbiz*$^{fl/fl}$*Vil1-Cre* mice compared to control mice, respectively. Open circles are peaks with a milder reduction (red) or increase (green) in *Nfkbiz*$^{fl/fl}$*Vil1-Cre* mice. No peaks were identified at *Defa30*, *Defa3*, *Defa5*, and *Mptx2* in (**b**) according to the criteria in the present study.

Supplementary Fig. 9

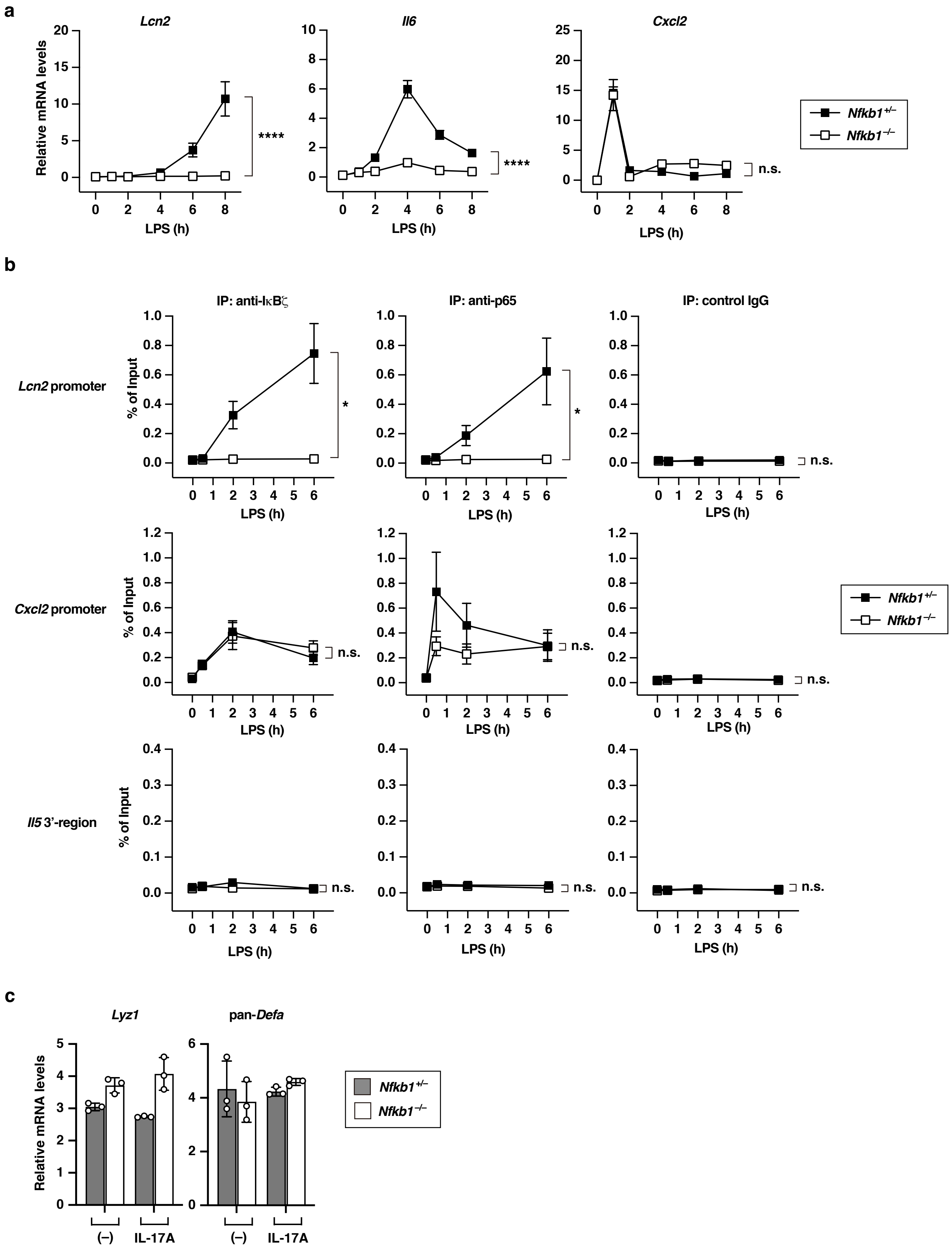

Supplementary Fig. 9　NF-κB p50 is required for IκBζ-mediated gene regulation.

a, b Bone marrow-derived macrophages from control (*Nfkb1*$^{+/-}$) or the NF-κB p50-deficient (*Nfkb1*$^{-/-}$) mice were stimulated with LPS (100 ng/ml) for the indicated periods. Total RNA was extracted, and the expression of the indicated genes was analyzed by RT-qPCR. The mean expression levels ± SEMs are shown (n=4 mice per group) (**a**). LPS-stimulated macrophages were subjected to chromatin immunoprecipitation (ChIP) using the indicated antibodies, and the occupancy of IκBζ or p65 at the indicated promoters was evaluated by qPCR. The mean values ± SEMs are shown (n=4 mice per group) (**b**). Statistical significance was determined by Two-way ANOVA (**a**, **b**). **p*<0.05, ****p<0.0001, n.s. not significant. **c** Small intestinal organoids from control (*Nfkb1*$^{+/-}$) or the NF-κB p50-deficient (*Nfkb1*$^{-/-}$) mice were stimulated with IL-17A (20 ng/ml) for 24 h. Expression of the indicated genes was analyzed by RT-qPCR. The results are presented as the mean ±SD of triplicates and are representative of organoids from three mice.

Supplementary Table 1: Sequences of *in situ* hybridization probes

Target	Probe	Probe sequence
SFB *16S rRNA*	First Probe	5'-CAGACTCCTCCTATACCAATAAATCaaACCTAAGGTTCA-3'
	Second Probe	5'-GCTCCTACGaaTCACTACATAATCATGCAACTATATAGCT-3'
Nfkbiz-1	First Probe	5'-CTGCGGACACTGCACTCTTCAGGTCaaGGCAAGTAGGAT-3'
	Second Probe	5'-CGGTGGAGTaaTCAGCTGCTCAATGTTCACACTCTG-3'
Nfkbiz-2	First Probe	5'-GAAGTGAGAAGGCAACAGAGCTGGCaaGGCAAGTAGGAT-3'
	Second Probe	5'-CGGTGGAGTaaCTTGCAAACCGGTCCATCGGACAGA-3'
Nfkbiz-3	First Probe	5'-CAGCGCTGCTATCCAGCTTGGATTCaaGGCAAGTAGGAT-3'
	Second Probe	5'-CGGTGGAGTaaTGTGGCGAACATCTTCCATGCTCTC-3'
Nfkbiz-4	First Probe	5'-GCTCATCCAGCTAACCTGAACAGTGaaGGCAAGTAGGAT-3'
	Second Probe	5'-CGGTGGAGTaaTAGTGAAACTGGGTTGCACTCATTC-3'
Nfkbiz-5	First Probe	5'-GTATTTCTGAGGTGGAGAGAAGGCCaaGGCAAGTAGGAT-3'
	Second Probe	5'-CGGTGGAGTaaGCCTCCATGGAAATCCTGGCACTGG-3'
Nfkbiz-6	First Probe	5'-GAATACTGGTACATTGACGCCTGGTaaGGCAAGTAGGAT-3'
	Second Probe	5'-CGGTGGAGTaaATCATCTGTGGAGAGCCACTGACTT-3'
Nfkbiz-7	First Probe	5'-GAATCATACTTGGGAGACTGGGACAaaGGCAAGTAGGAT-3'
	Second Probe	5'-CGGTGGAGTaaCTGGAGTAAGGACTGAACTGCAGGG-3'
Nfkbiz-8	First Probe	5'-CAGGCATTGCTTGGCATCAGGCTGAaaGGCAAGTAGGAT-3'
	Second Probe	5'-CGGTGGAGTaaGTCTGCAGCTGAGTCTCAGTTTGGG-3'
Nfkbiz-9	First Probe	5'-GTATTCATCGGGCTTCCCATGATGTaaGGCAAGTAGGAT-3'
	Second Probe	5'-CGGTGGAGTaaTGGAGTGACAGGGACGTGCCCAAAG-3'
Vil1-1	First Probe	5'-CTCGATCCTCCATATCTGTATCCCGaaTCCATCTAAGCT-3'
	Second Probe	5'-CCTCCACGTaaAGTGGTGATGTTGAGAGAGCCTTTG-3'
Vil1-2	First Probe	5'-ACCATCGAAGAAGCTTCCAAAGGTGaaTCCATCTAAGCT-3'
	Second Probe	5'-CCTCCACGTaaGGAAGGAACAGGTACCATCTGCATA-3'
Vil1-3	First Probe	5'-GTCTCGGATCTCTTTGGCCAAGGCCaaTCCATCTAAGCT-3'
	Second Probe	5'-CCTCCACGTaaGCCCCGAAGTCTCTCCATGCGGTTA-3'
Vil1-4	First Probe	5'-CACCAGTTTTCCTTCGGAGTCAGACaaTCCATCTAAGCT-3'
	Second Probe	5'-CCTCCACGTaaATGGTACAGCTTGAGTGCAGCCTTA-3'
Vil1-5	First Probe	5'-CCGTCATTCTGCACCTCAACCTGCGaaTCCATCTAAGCT-3'
	Second Probe	5'-CCTCCACGTaaCTCGGTGGGTACTGCTTGGCTTTGA-3'

Vil1-6	First Probe	5'-CACCTGTTCCACCTTAGCCACAGAGaaTCCATCTAAGCT-3'
	Second Probe	5'-CCTCCACGTaaCACAGTGTGGGTTTTGCCGAGGCCT-3'
Vil1-7	First Probe	5'-TTTCTGCTGGGCAGCCACCTGAGGTaaTCCATCTAAGCT-3'
	Second Probe	5'-CCTCCACGTaaTACATGCATGGTCAGAGCATCAAAC-3'
Vil1-8	First Probe	5'-CGGATCTGTACTGGCTCGTCATTGTaaTCCATCTAAGCT-3'
	Second Probe	5'-CCTCCACGTaaTTCTGGTCCAACAGGACGGCTTGAT-3'
Vil1-9	First Probe	5'-AAGTTGTTCTTTCGGGAGGTGCCTCaaTCCATCTAAGCT-3'
	Second Probe	5'-CCTCCACGTaaTGATAAACCACCATGCGGCCCTTGA-3'
Vil1-10	First Probe	5'-CACTTGTTTCTCCGTCCGAGAGATGaaTCCATCTAAGCT-3'
	Second Probe	5'-CCTCCACGTaaATCAGCAACCATCTTGGCCATCTCC-3'
Vil1-11	First Probe	5'-TTGTACAGTTGTAGCTGCAGCCTTCaaTCCATCTAAGCT-3'
	Second Probe	5'-CCTCCACGTaaCTCTTCCTCATTGGCATGTTTCCCT-3'
Vil1-12	First Probe	5'-TTCACCACGATGATAGGGGTCTCAAaaTCCATCTAAGCT-3'
	Second Probe	5'-CCTCCACGTaaTCTCGGTTTCCAGGGTGGGTCTTGA-3'
Vil1-13	First Probe	5'-GGGCTCATAACCTCGTCAGCAATCTaaTCCATCTAAGCT-3'
	Second Probe	5'-CCTCCACGTaaCTCCAGTCCCCAGAGTTTCCCAGCT-3'
Vil1-14	First Probe	5'-TCCACACCCTCAGGGAGATCCTCTAaaTCCATCTAAGCT-3'
	Second Probe	5'-CCTCCACGTaaGACTTGTTTACCAGCTGCTCCAGGG-3'
Vil1-15	First Probe	5'-GCTGGAGTCATGCCCAAGGCCCTAGaaTCCATCTAAGCT-3'
	Second Probe	5'-CCTCCACGTaaAAGTCTTCGGTGGACAGGTGCTCCT-3'
Lact-1	First Probe	5'-TCACCAGCTACCAAATCAGAGCCCTaaTGGATTGAGTGT-3'
	Second Probe	5'-GCTGGTCGGaaTTTCCCAGCGGATGGTTCAGATTGT-3'
Lact-2	First Probe	5'-GGTCTGGATTCTTGGAGCTTCCTTTaaTGGATTGAGTGT-3'
	Second Probe	5'-GCTGGTCGGaaGGAGAAGTTGTGCCCATGACAGAAG-3'
Lact-3	First Probe	5'-GCATCGCTGAGGGTTTGGAGACCAGaaTGGATTGAGTGT-3'
	Second Probe	5'-GCTGGTCGGaaGCTTTCAAATGCTGGTGGGGAAGAC-3'
Lact-4	First Probe	5'-GGACCACAGACAGCTTTCCTCCCTGaaTGGATTGAGTGT-3'
	Second Probe	5'-GCTGGTCGGaaAAGAGTACTTTCTGTGGTAAACGTC-3'
Lact-5	First Probe	5'-GCTCATTCCAGTCGATGGGCAGTCCaaTGGATTGAGTGT-3'
	Second Probe	5'-GCTGGTCGGaaAGGTTTTAGGGTGTAGACGAAAACT-3'
Lact-6	First Probe	5'-CCAGGCTACTGTAGGTTAAAGAACAaaTGGATTGAGTGT-3'
	Second Probe	5'-GCTGGTCGGaaATGGGCTCTCCTCAGAACTGGACGT-3'
Lact-7	First Probe	5'-ACCCTTCTGGAAATACATCTTGCAGaaTGGATTGAGTGT-3'
	Second Probe	5'-GCTGGTCGGaaAAGCGTCCCTTTCCTCCCTAGACTG-3'

Lact-8	First Probe	5'-TGCTGCTTTTGGCGATGGTGATAGTaaTGGATTGAGTGT-3'
	Second Probe	5'-GCTGGTCGGaaTAGTGATGCCAAGTTCTGGCATGCG-3'
Lact-9	First Probe	5'-TAGTCCCCATCTATGAAGATGGGGTaaTGGATTGAGTGT-3'
	Second Probe	5'-GCTGGTCGGaaGCAAACCAGCCTAGCATGAAGTGCA-3'
Lact-10	First Probe	5'-CATAGCCATCAATGAGGGAACGAGCaaTGGATTGAGTGT-3'
	Second Probe	5'-GCTGGTCGGaaTGTATGAACGGACATCCACTGAATC-3'
Slc10a2-1	First Probe	5'-GAATTGCATTGAAGTTGCTCTCAGGaaCCGTAAGTCAGA-3'
	Second Probe	5'-CCCTCCACAaaCTACGCAGGAATCGCCTTCGCAGAC-3'
Slc10a2-2	First Probe	5'-ACATTGCACCCCATAGAAAACATCAaaCCGTAAGTCAGA-3'
	Second Probe	5'-CCCTCCACAaaATGGCTAAGAGGATGGTGAGCACAG-3'
Slc10a2-3	First Probe	5'-ATAATTAGCACCACTACAGCCTGTAaaCCGTAAGTCAGA-3'
	Second Probe	5'-CCCTCCACAaaGGAAGGATGCCAGAGGCCACAGACA-3'
Slc10a2-4	First Probe	5'-ACTGAGGTCCATGTCGCCATCTATCaaCCGTAAGTCAGA-3'
	Second Probe	5'-CCCTCCACAaaATAGGCCAGGATATTGGAGCCAGTT-3'
Slc10a2-5	First Probe	5'-ATTCCAGTTTCCAAGGCTACTGTTCaaCCGTAAGTCAGA-3'
	Second Probe	5'-CCCTCCACAaaCACCTGTACCAGGGTTGACCAGCTA-3'
Cre-1	First Probe	5'-CCCGGACCGACGATGAAGCATGTTTaaGGGTTCAGTCTA-3'
	Second Probe	5'-CGTCGGAGTaaCTGGCCCAAATGTTGCTGGATAGTT-3'
Cre-2	First Probe	5'-TGAGTGAACGAACCTGGTCGAAATCaaGGGTTCAGTCTA-3'
	Second Probe	5'-CGTCGGAGTaaTGCGTTCGAACGCTAGAGCCTGTTT-3'
Cre-3	First Probe	5'-ACCCTGATCCTGGCAATTTCGGCTAaaGGGTTCAGTCTA-3'
	Second Probe	5'-CGTCGGAGTaaCGTAACAGGGTGTTATAAGCAATCC-3'
Cre-4	First Probe	5'-ATCATCAGCTACACCAGAGACGGAAaaGGGTTCAGTCTA-3'
	Second Probe	5'-CGTCGGAGTaaCCATCGCTCGACCAGTTTAGTTACC-3'
Cre-5	First Probe	5'-CTCTGACCAGAGTCATCCTTAGCGCaaGGGTTCAGTCTA-3'
	Second Probe	5'-CGTCGGAGTaaTAAATCAATCGATGAGTTGCTTCAA-3'
Lyz1-1	First Probe	5'-CAGAAAGCAGGAGGAGTCCCAGAGTaaAGCCCATTAGAT-3'
	Second Probe	5'-CGTCGGATGaaGGAGAGCCTTCATGGTGACTGGAGG-3'
Lyz1-2	First Probe	5'-CTTTTCAGAATTCTGGCCAACTCACaaAGCCCATTAGAT-3'
	Second Probe	5'-CGTCGGATGaaCGATTGTAGACCTTGGCCTGGGCAG-3'
Lyz1-3	First Probe	5'-TAATTGCTCTCATGCTGAGCTAAACaaAGCCCATTAGAT-3'
	Second Probe	5'-CGTCGGATGaaACCCAGTCAGCCAGCTTGACACCAC-3'
Lyz1-4	First Probe	5'-CGGCTATTGATCTGAAATATCCCATaaAGCCCATTAGAT-3'
	Second Probe	5'-CGTCGGATGaaTCGGTGCTTCGGTCTCCACGGTTGT-3'

Lyz1-5	First Probe	5'-GAGCACTGCAATTGATCCCACAGGCaaAGCCCATTAGAT-3'
	Second Probe	5'-CGTCGGATGaaTCTTAGATCTTGGGGTTTTGCCATC-3'
Lyz1-6	First Probe	5'-AATGCCTTGGGGATCTCTCACCACCaaAGCCCATTAGAT-3'
	Second Probe	5'-CGTCGGATGaaCTTTGCACATTGTATGGCTGCAGTG-3'
Lyz1-7	First Probe	5'-CTCCGCAGTTCCGAATATACTGGGAaaAGCCCATTAGAT-3'
	Second Probe	5'-CGTCGGATGaaGATCTCGGTTTTGACATTGTGTTCG-3'
Lyz1-8	First Probe	5'-CTCTCTCATATCTACTCCTACAGTGaaAGCCCATTAGAT-3'
	Second Probe	5'-CGTCGGATGaaAAAGAGACAGAATGGGCTGCAGTAG-3'
Lyz1-9	First Probe	5'-AGGAAGTGTCTGTTTTGCCCTGTTTaaAGCCCATTAGAT-3'
	Second Probe	5'-CGTCGGATGaaGCTGAAGTCCTGTTACTTGGAGGGG-3'
Lyz1-10	First Probe	5'-CAGCATCCATGGCTTTGCTGACTGAaaAGCCCATTAGAT-3'
	Second Probe	5'-CGTCGGATGaaAGGGAGACTTTGCACAACACAGCAT-3'
Lyz1-11	First Probe	5'-TCACTTCCTGGCTGAAGAACTGACCaaAGCCCATTAGAT-3'
	Second Probe	5'-CGTCGGATGaaCAGAGATGTCTGTGTGGCAATCACT-3'
Lyz1-12	First Probe	5'-CTGGGTGCTGGGACTGCACAAAGCCaaAGCCCATTAGAT-3'
	Second Probe	5'-CGTCGGATGaaGCAGCTCACACAACTGCTGTTTTCC-3'
Lyz1-13	First Probe	5'-ACAATCTCAGTTCTCATCCACTGAGaaAGCCCATTAGAT-3'
	Second Probe	5'-CGTCGGATGaaTCCTGTGCCCTCAGAAACCCTCCCG-3'
Lyz1-14	First Probe	5'-TCATGACACTGGGAACATCCTCTCAaaAGCCCATTAGAT-3'
	Second Probe	5'-CGTCGGATGaaGGTTAAACAGACTGACACATGAGCT-3'

Supplementary Table 2: Sequences of hairpin DNAs for *in situ* hybridization

Hairpin		Hairpin sequence	Label
S10	H1	5'-ATCTAATGGGCTCATCCGACGATCACTTGACGTCGGATGAGC-3'	SaraFluor488
	H2	5'-CGTCGGATGAGCCCATTAGATGCTCATCCGACGTCAAGTGAT-3'	
S25	H1	5'-TAGACTGAACCCACTCCGACGATCTGTCTTCGTCGGAGTGGG-3'	Alexa647
	H2	5'-CGTCGGAGTGGGTTCAGTCTACCCACTCCGACGAAGACAGAT-3'	
S40	H1	5'-TGAACCTTAGGTCGTAGGAGCACTTTCACTGCTCCTACGACC-3'	Alexa647
	H2	5'-GCTCCTACGACCTAAGGTTCAGGTCGTAGGAGCAGTGAAAGT-3'	
S45	H1	5'-AGCTTAGATGGAACGTGGAGGTAACAAGCACCTCCACGTTCC-3'	SaraFluor488
	H2	5'-CCTCCACGTTCCATCTAAGCTGGAACGTGGAGGTGCTTGTTA-3'	
S72	H1	5'-ATCCTACTTGCCACTCCACCGTTGAGTTGACGGTGGAGTGGC-3'	ATTO550
	H2	5'-CGGTGGAGTGGCAAGTAGGATGCCACTCCACCGTCAACTCAA-3'	
S83	H1	5'-TCTGACTTACGGTGTGGAGGGTCTTAACCACCCTCCACACCG-3'	SaraFluor488
	H2	5'-CCCTCCACACCGTAAGTCAGACGGTGTGGAGGGTGGTTAAGA-3'	
S86	H1	5'-ACACTCAATCCACCGACCAGCACAAGTCATGCTGGTCGGTGG-3'	ATTO550
	H2	5'-GCTGGTCGGTGGATTGAGTGTCCACCGACCAGCATGACTTGT-3'	

Supplementary Table 3: Sequences of qPCR Primers

Analysis on mRNA expression

Gene	Primer #1	Primer #2
Nfkbiz	5'-ATGGCCCAGTGGGAGAACA-3'	5'-CAGTGGACCTAGTATGGTGGT-3'
Pigr	5'-GATCTGTCTTCAAGACCTCTGC-3'	5'-CTTAATATTGGGATTGCAGGAGC-3'
Foxp3	5'-GGCGAAAGTGGCAGAGAGG-3'	5'-AAGGCAGAGTCAGGAGAAGTTG-3'
Ccl28	5'-TCTTATAAAGCCACTAGCATCC-3'	5'-GTATCCTTAAATCCATATGTTGG-3'
Cd177	5'-CACTGTTATAAAGGTGACATTGC-3'	5'-TACCGATTGTTTTGGAGTCACC-3'
Defa4	5'-CCAGGGGAAGATGACCAGGCTG-3'	5'-TGCAGCGACGATTTCTACAAAGGC-3'
Defa31	5'-AGGCTGCAAAAGAAGAGAACG-3'	5'-GTCTTGTCCTCTGTGGTCTC-3'
Itln1	5'-ATGGATATGGAACTCACAATGG-3'	5'-GGAGAAGTCAGGGCCAATCC-3'
Il17a	5'-CAGACTACCTCAACCGTTCCAC-3'	5'-TCCAGCTTTCCCTCCGCATTGA-3'
Il17f	5'-CAAAACCAGGGCATTTCTGT-3'	5'-ATGGTGCTGTCTTCCTGACC-3'
Lyz1	5'-AGCTGCCCCTTTCATCTTGC-3'	5'-CAGGCACAGCTCACTAGTCC-3'
pan-Defa	5'-GGTGATCATCAGACCCCAGCATCAGT-3'	5'-AAGAGACTAAAACTGAGGAGCAGC-3'
Igha	5'-GACCGTCTGTCGGGTAAACC-3'	5'-TCTTCTGACTGGTCCAGTAGC-3'
Il22	5'-GCTCCTGTCACATCAGCGGTGAC-3'	5'-GCAGGTCCAGTTCCCCAATCGCC-3'
Reg3b	5'-TGACATGTGAGGTGAAGTTGC-3"	5'-CTTCACATTTTGTCCCTTGTCC-3'
Reg3g	5'-ACGAATCCTTCCTCTTCCTCAG-3'	5'-GTCTTCACATTTGGGATCTTGC-3'
Muc2	5'-ACATCACCTGTCCCGACTTC-3'	5'-GAGCAAGGGACTCTGGTCTG-3'
Vil1	5'-CTATGCAGATGGTACCTGTTC-3'	5'-CCTGGGACGAGTCCTGGCCAA-3'
H2-Aa	5'-TGTTTTCTGGACCAATTCATGG-3'	5'-GATCAGGGAAGAATTCCAAGG-3'
Lgr5	5'-CGTAGGCAACCCTTCTCTTATC-3'	5'-GCACCATTCAAAGTCAGTGTTC-3'
Lcn2	5'-AAGGAGCTGTCCCCTGAACT-3'	5'-GGTGGGGACAGAGAAGATGA-3'
Il6	5'-CACAGAGGATACCACTCCCAA-3'	5'-TCCACGATTTCCCAGAGAACA-3'
Cxcl2	5'-GGATTTCAATGTAATGTTGTGAG-3'	5'-CAAAACAATTGCTAAGCAAGGC-3'
Hprt	5'-GCAGTACAGCCCCAAAATGG-3'	5'-AACAAAGTCTGGCCTGTATCCAA-3'
Ifng	5'-CAGCAACAGCAAGGCGAAAAAGG-3'	5'-TTTCCGCTTCCTGAGGCTGGAT-3'
Ciita	5'-ACCTTCGTCAGACTGGCGTTGA-3'	5'-GCCATTGTATCACTCAAGGAGGC-3'
Il1b	5'-TGGACCTTCCAGGATGAGGACA-3'	5'-GTTCATCTCGGAGCCTGTAGTG-3'
Tnf	5'-GGTGCCTATGTCTCAGCCTCTT-3'	5'-GCCATAGAACTGATGAGAGGGAG-3'
Il23a	5'-CATGCTAGCCTGGAACGCACAT-3'	5'-ACTGGCTGTTGTCCTTGAGTCC-3'

Analysis on abundance of bacteria

Target	Primer #1	Primer #2
SFB	5'-GACGCTGAGGCATGAGAGCAT-3'	5'-GACGGCACGGATTGTTATTCA-3'
Eubacteria	5'-ACTCCTACGGGAGGCAGCAGT-3'	5'-ATTACCGCGGCTGCTGGC-3'
Actb	5'-GGCTGTATTCCCCTCCATCG-3'	5'-CCAGTTGGTAACAATGCCATGT-3'

ChIP analysis

Target	Primer #1	Primer #2
Lcn2 promoter	5'-GGGGAGAGAGGGACAGAAAT-3'	5'-CCTTTACCAAGTCCAGGAAGC-3'
Cxcl2 promoter	5'-TCCCGAGAGCTCCTTTTATG-3'	5'-GGGCTCTGTGCTTCCTGAT-3'
Il5 3'-region	5'-AAGGGTGGACTTGGTAGTGG-3'	5'-GAGCCATCTGGACATACTCC-3'
